# Zyflamend induces apoptosis in pancreatic cancer cells via modulation of the JNK pathway

**DOI:** 10.1186/s12964-020-00609-7

**Published:** 2020-08-14

**Authors:** Dexter L. Puckett, Mohammed Alquraishi, Dina Alani, Samah Chahed, Dallas Donohoe, Brynn Voy, Jay Whelan, Ahmed Bettaieb

**Affiliations:** 1 Department of Nutrition, University of Tennessee Knoxville, 1215 Cumberland Avenue, 229 Jessie Harris Building, Knoxville, TN 37996-0840 USA; 2 Tennessee Agricultural Experiment Station, University of Tennessee Institute of Agriculture, Knoxville, TN 37996-0840 USA; 3 Graduate School of Genome Science and Technology, University of Tennessee, Knoxville, TN 37996-0840 USA; 4 Department of Biochemistry, Cellular and Molecular Biology, University of Tennessee, Knoxville, TN 37996-0840 USA

**Keywords:** Pancreatic neuroendocrine tumor cells, Zyflamend, JNK, Apoptosis, Autophagy, ER stress

## Abstract

**Background:**

Current pharmacological therapies and treatments targeting pancreatic neuroendocrine tumors (PNETs) have proven ineffective, far too often. Therefore, there is an urgent need for alternative therapeutic approaches. Zyflamend, a combination of anti-inflammatory herbal extracts, that has proven to be effective in various in vitro and in vivo cancer platforms, shows promise. However, its effects on pancreatic cancer, in particular, remain largely unexplored.

**Methods:**

In the current study, we investigated the effects of Zyflamend on the survival of beta-TC-6 pancreatic insulinoma cells (β-TC6) and conducted a detailed analysis of the underlying molecular mechanisms.

**Results:**

Herein, we demonstrate that Zyflamend treatment decreased cell proliferation in a dose-dependent manner, concomitant with increased apoptotic cell death and cell cycle arrest at the G2/M phase. At the molecular level, treatment with Zyflamend led to the induction of ER stress, autophagy, and the activation of c-Jun N-terminal kinase (JNK) pathway. Notably, pharmacological inhibition of JNK abrogated the pro-apoptotic effects of Zyflamend. Furthermore, Zyflamend exacerbated the effects of streptozotocin and adriamycin-induced ER stress, autophagy, and apoptosis.

**Conclusion:**

The current study identifies Zyflamend as a potential novel adjuvant in the treatment of pancreatic cancer via modulation of the JNK pathway.

**Video abstract**

## Plain English summary

Through investigating the effects of treating an experimental model of pancreatic neuroendocrine tumor cells with Zyflamend, we discovered a novel therapeutic potential of this polyherbal blend. Findings from this study could help pioneer future advancements in our understanding of how phytochemicals and natural compounds could synergistically prove effective against pancreatic cancer by altering cancer cell survival and proliferation. Furthermore, the evidence presented within promotes Zyflamend as an adjuvant prospect, where it could enhance the effectiveness of standard cancer therapies. In addition, we believe that these novel findings will be of major interest to a broad spectrum of scientists, and may pave the way towards more effective and translatable therapies.

## Background

Pancreatic cancer remains one of the deadliest types of cancer in the United States with over 56,770 new cases and 45,750 deaths in 2019, accounting for 7.5% of all cancer deaths [1]. Recent epidemiological studies predict that in 2019, pancreatic cancer will be the third leading cause of cancer-related death [1]. Although advancements in science and health care have led to decreased mortality from numerous forms of cancer, pancreatic cancer survival rates have not improved significantly over the past several decades, leaving a desperate need for more effective treatment options. The risk of developing pancreatic cancer has been associated with numerous biological, environmental, pathological, and genetic factors. These factors include variables such as familial history, chronic pancreatitis, smoking, obesity, and diabetes (reviewed in [2, 3]). In addition, hereditary familial factors and germline mutations could contribute to increased risk of cancer onset. However, the survival outcome and treatment effectiveness are highly dependent upon the time of diagnosis. While pancreatic ductal adenocarcinoma (PDAC) is the most commonly contracted and investigated subtype, the pathological nature of the rarer pancreatic neuroendocrine tumors (PNETSs) remains elusive [4].

PNETs account for less than 5% of all pancreatic cancers and are often diagnosed at a late stage in patients with advanced metastasis making surgery a non-viable treatment option [5, 6]. Additionally, because of their heterogeneous clinical presentation and responses to chemotherapeutic agents, current pharmacological therapies and treatment options targeting PNETs have too often proven in-effective [4]. PNETs treatment options often include the use of chemotherapeutic compounds such as streptozocin, 5-fluorouracil, doxorubicin, and cisplatin, both alone or in combination (reviewed in [6, 7]). The effectiveness of these compounds often increases at higher doses, but this directly exacerbates the risk for cytotoxicity and collateral side effects [8]. In addition, adjunct therapy involving the combination of various treatment approaches, such as surgery and radiotherapy, is often implemented [9]. In pursuit of survival and improved quality of life, patients often seek to enhance the effectiveness of conventional therapies through dietary and supplemental means [10, 11].

New Chapter (Brattleboro, VT) first launched Zyflamend based on the idea of combining extracts of ten different herbs to effectively reduce inflammation through cyclooxygenase (COX) inhibition [12]. A large volume of research has emerged over the last two decades that supports the anti-inflammatory properties of Zyflamend and its ability to inhibit COX in various types of cancer, including prostate [13], melanoma [14], and oral cancer [15]. Individually, many of the extracted components of Zyflamend have proven to exhibit anti-cancer activity [16, 17]. However, the high doses required to optimize effectiveness against cancer could prove infeasible for the majority. In theory, the combined effects generated through integrating these unique and powerful herbs could grant superior benefit over their isolated form [18]. Additionally, Zyflamend has shown the capability to interact with a variety of integral cellular signaling pathways beyond COX. These signaling pathways and mechanisms of interaction include: AMP-activated protein kinase (AMPK) [19, 20], nuclear factor kappa-light-chain-enhancer of activated B cells (NF-κB) [21, 22], mammalian target of rapamycin (mTORC1) [20], apoptosis, cell growth [13, 23], endoplasmic reticulum (ER) stress [22, 24, 25] and finally autophagy [14]. While these studies show that Zyflamend could exhibit profound potential in the therapeutic application, more research is required to elucidate the molecular basis underlying its anti-cancer effects. In the current study, we investigated the effects of Zyflamend on the survival of beta-TC-6 pancreatic insulinoma cells (β-TC6) and deciphered the underlying molecular mechanisms.

## Methods

### Chemicals and reagents

Media, sera, and trypsin for cell culture were purchased from Gibco (Thermo Fisher Scientific, Waltham, MA). Primary antibodies and secondary antibodies were acquired from varying sources (Supplementary Table 1). General caspases inhibitor (Z-VAD.fmk) was obtained from Calbiochem (La Jolla, CA). Zyflamend™whole body was purchased from New Chapter (New Chapter, Inc. Brattleboro, VT). Zyflamend composition is indicated in Supplementary Table 2. Quality assurance is in full compliance with Good Manufacturing Practicing Standards as mandated by 21 CRF Part 111. Additionally, full description and characterization of Zyflamend and its preparation have been previously described in detail [26]. Chemical reagents such as dithiothreitol (DTT), percoll, digitonin, phenylmethylsulfonyl fluoride (PMSF), protease inhibitors cocktail, sodium deoxycholate, Triton X-100, ethylene glycol-bis-(2-aminoethyl)-N,N,N′,N′-tetraacetic acid (EGTA), sodium fluoride (NaF), sodium phenylbutyrate (4-PBA), Hoechst 33258, propidium iodide, streptozotocin (STZ), adriamycin (ADR), autophagy inhibitor 3-methyladenine (3-MA), and JNK inhibitor (SP600125) were acquired from Millipore-Sigma (Burlington, MA). Finally, AMPK inhibitor (BML-275; a.k.a. compound C) was purchased from Santa Cruz Biotechnology (Santa Cruz, CA).

### Cell culture

Mouse beta-TC-6 pancreatic insulinoma (β-TC6; ATCC® CRL-11506™) and rat pancreatic insulinoma RIN-5F (ATCC® CRL-2058™) cells were cultured as monolayers in Eagle’s modified Dulbecco medium plus L-glutamine (2 mM), sodium pyruvate (1 mM) and 10% fetal bovine serum (FBS) (Gibco-Thermo Fisher Scientific, Waltham, MA). Cells were maintained in tissue culture plates (Thermo Fisher Scientific, Waltham, MA) at 37 °C in a humidified atmosphere of 10% CO_2_. Medium was replaced with fresh medium 24 h before experiments.

### Zyflamend treatment

Zyflamend was dissolved in dimethyl sulfoxide (DMSO) at a concentration of 800 mg/ml. Cells were treated with Zyflamend at the indicated concentrations and for the indicated durations. Treatments were terminated by two washes with ice-cold phosphate buffer saline (PBS). Plates were then flash-frozen in liquid nitrogen and stored at − 80 °C until further analyses.

### Proliferation assay

Cell proliferation assay was performed using the sulforhodamine B (SRB; Millipore-Sigma) method as previously described [27] with modification. Briefly, an equal number of β-TC6 cells (1 X 10^6^ cells) were seeded in 6 well plates. Six h. later cells were treated with the indicated concentrations of Zyflamend and incubated at 37 °C in an atmosphere of 10% CO_2_ for the indicated time. Treatment was stopped by two washes with ice cold PBS and cells were fixed with 17% trichloroacetic acid in PBS. Intracellular proteins were stained for 10 min at room temperature using 0.4% SRB dissolved in 1% acetic acid. Excess SRB stain was removed by rinsing the plates thoroughly with running tap water. Plates were air-dried for at least 6 h prior to dissolving the stain in 10 mM Tris (pH 9.0). Intracellular proteins were quantified using the Synergy™ HTX Multi-Mode microplate reader (BioTek Instruments, Inc. Winooski, VT) at a wavelength of 540 nm. The relative survival rates of cells were determined by dividing the absorbance observed for a given treatment by the absorbance detected in control cells treated with DMSO, and expressed as a fold change.

### Cytotoxicity assay

The MTT (3-[4,5-dimethylthiazol-2-yl]-2,5-diphenyltetrazolium bromide) cytotoxicity assay was performed as previously described with modification [28]. Briefly, 10,000 cells were plated in a 96-well plate for 24 h. Then, a freshly prepared solution of Zyflamend alone or in combination with Z-VAD.fmk (10uM), 4-PBA (250uM), SP600125 (10uM), STZ (5 mM) or adriamycin (5 μM) for an additional 24 h. The experiment was terminated by adding 40 μl of the MTT solution (5 mg/ml) to each well for 4 h, then the cell culture medium was removed and the dye was dissolved in 100 μl SDS solution (10%) overnight at 37 °C. Relative cytotoxicity was determined by measuring the absorbance at 570 nm using the Synergy™ HTX Multi-Mode microplate reader.

### Colognic test

Colonie formation assay was performed as previously described [29, 30] with modification. Cells were seeded in the presence of DMSO (control), Zyflamend alone or in combination with Z-VAD.fmk (10 uM), 4-PBA (250 uM), SP600125 (10 uM), STZ (5 mM) or adriamycin (5 μM). After 24 h, media was replaced with a freshly prepared new cell culture media and plates were incubated for 7 days at 37 °C in an atmosphere of 10% CO_2_. After incubation, the colonies were washed with ice-cold PBS, fixed and stained with a mixture of 6.0% glutaraldehyde and 0.5% crystal violet for 30 min. The plates were washed with water, dried, and colonies (with more than 30 cells/colony) were counted. The relative number of colonies in each condition was determined by dividing the number of colonies for a given treatment by the total number of colonies in DMSO treated cells (control), and expressed as a percentage relative to DMSO-treated cells (Ctrl.).

### Western blotting analysis

Cells were lysed in radio-immunoprecipitation assay (RIPA) buffer, as previously described [31]. Lysates were clarified by centrifugation at 15,000 g for 10 min, and protein concentrations was determined using bicinchoninic acid assay kit (Pierce Chemical, Dallas, TX). Proteins (5–30 μg) were resolved by sodium dodecyl sulfate polyacrylamide gel electrophoresis (SDS-PAGE) and transferred to polyvinylidene fluoride (PVDF) membranes. Immunoblotting of lysates was performed with primary antibodies (Supplementary Table 1), and after incubation with secondary antibodies, proteins were visualized using Luminata™ Forte Western Chemiluminescent HRP Substrate (Millipore-Sigma). Pixel intensities of immunoreactive bands were quantified using FluorChem Q Imaging software (Alpha Innotech Corp., San Leandro, CA). Data for phosphorylated proteins are presented as the intensity of phosphorylation normalized to total protein expression, while total protein expression was normalized to the loading control: β-actin.

### Morphological analysis of apoptosis

β-TC6 cells were exposed to Zyflamend for the indicated duration then washed with PBS and labeled with Hoechst 33258 (50 μg/ml in PBS) (blue-green fluorescence). Hoechst binds to condensed nuclear chromatin [31] and was used to visualize apoptotic cells (green fluorescence) by fluorescence microscopy (Leica DMI8, Leica Microsystems Inc. Buffalo Grove, IL). For each condition, at least 500 cells were counted. Percentages of apoptotic cells were calculated relative to total cells.

### Annexin V staining

Quantification of externalized phosphatidylserine, an early event in the apoptotic cascade, was performed using flow cytometry as previously described [32] with modification. Briefly, 50–60% confluent β-TC6 cells were exposed to Zyflamend for 24 h then washed with PBS and resuspended in 200 μl of PBS containing 10% FBS. Immediately after, an equal volume of the 2X Guava Nexin reagent (Millipore-Sigma) containing Annexin V- Fluorescein isothiocyanate (FITC) and 7-amino-actinomycin D (7-AAD) was added to each treatment and incubated for 10 min at room temperature under light-protected conditions. Intensities of fluorescence emitted by Annexin V- FITC and 7-AAD were measured using the Guava® easyCyte Flow Cytometer (Millipore-Sigma) on PM1 and PM2 channels, respectively. Viable (negative for both Annexin V and 7-AAD staining) and apoptotic cells, both at early (Annexin V positive, 7-AAD negative) and late (positive for both Annexin V and 7AAD) stages were quantified using the InCyte™ and GuavaSuite Software package (Luminex Corp; Austin, TX).

### Cell cycle analysis

Cell cycle analysis was conducted through assessing the DNA content of cells stained with propidium iodide as previously described [33] with modification. Briefly, 50–60% confluent β-TC6 cells were starved in 0.1% serum media for 16 h, then complete growth media was added to the cells along with various freshly prepared concentrations of Zyflamend. 24 h later, cells were harvested, washed twice with ice-cold with PBS, and fixed overnight in 70% ethanol at 4 °C. Next, cells were washed twice with ice-cold PBS and incubated in a freshly prepared RNase solution [10 mM Tris-HCl, pH 7.4 containing 100 U/ml of DNase-free RNase A (Applied Biosystems, Austin, TX)] for 30 min at 37 °C. Cells were washed twice with ice-cold PBS and incubated in a solution of propidium iodide (PI; 10 μg/ml in PBS) overnight at 4 °C under light protected conditions. Fluorescence intensity of PI was measured using the Guava® easyCyte flow cytometer on PM2 channel. DNA histogram analysis was performed on 5000 cells using the InCyte™ and GuavaSuite Software package and the proportions of cells with one or two copies of their chromosomal DNA were calculated.

### Statistical analysis

Data were analyzed using JMP Pro 14.2 program (SAS Institute, NC) and presented as means + standard error of the mean (SEM). Unpaired heteroscedastic two-tail Student’s t test was used for all statistical analyses, and differences were considered significant at *p* < 0.05. Single symbol (such as * or †) was used to indicate a *p* value that is less than 0.05, while double symbol (such as ** or ††) corresponds to a *p* value that is less than 0.01.

## Results

### Zyflamend decreases cell proliferation, causes G2/M cell-cycle arrest, and induces apoptotic cell death in pancreatic cancer cells

**Fig. 1:**
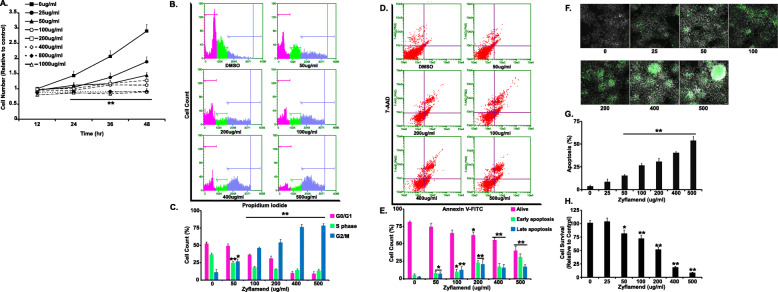
Zyflamend Reduces Cell Survival and Induces Cell Death of Pancreatic Cancer Cells in a Dose Dependent Manner. **a** Effects of Zyflamend on cell survival and proliferation: cells were treated with increasing doses of Zyflamend for 24 h. Line graphs represent the intensity of SRB staining reflective of the cell number and presented as means + SEM. **b-c** Cell cycle analysis and assessment of DNA content in β-TC6 cells treated with DMSO (control) or the indicated concentration of Zyflamend for 24 h. Representative histogram distributions for each treatment are shown. **c** Bar graphs represent the percentages of cells in each phase of the cell cycle, which were estimated using the GuavaSuite Software package and are presented as means + SEM from three independent experiments. *p < 0.05, **p < 0.01 indicate significant difference between the indicated concentration and control cells treated with the vehicle DMSO. **d-e** Zyflamend treatment induces apoptosis in β-TC6 Cells: 50% confluent cells were treated with increasing concentrations of Zyflamend, and then labeled with Annexin V-FITC and 7-AAD. Representative dot plots are shown. Annexin V positive and /7-AAD negative cells (lower right quadrants) represent early stages of apoptosis, whereas cells that are positive for both Annexin V and 7-AAD (upper right quadrants) are in late stages of apoptosis. **e** Bar graphs represent live, early, and late apoptotic cells are presented as means + SEM of at least three independent experiments. *p < 0.05, **p < 0.01 indicate significant difference between the indicated concentration of Zyflamend and control cells treated with the vehicle DMSO. **f-g** Chromatin condensation in cells treated with increasing doses of Zyflamend for 24 h. Representative images are shown. Scale bar: 50 μm. **g** Bar graphs represent the number of apoptotic cells (Hoechst positive) as means + SEM. of at least three independent experiments. **h** Cell toxicity assay using the MTT method. Bar graphs represent the intensity of formazan (produced from MTT by viable cells) staining reflective of the cell number and presented as means + SEM. of at least three independent experiments. In **g** and **h** *p < 0.05, **p < 0.01 indicate a significant difference between cells treated with Zyflamend and non-treated cells

We first examined the effects of varying doses of Zyflamend on the proliferation of pancreatic insulinoma β-TC6 cells. Zyflamend caused a significant dose- and time-dependent decrease in cell growth (Fig. 1a). Additionally, a Zyflamend dose of 25 μg/ml was sufficient to inhibit cell proliferation by 58% after 36 h of treatment, while a dose of 800 μg/ml completely abolished cell proliferation (Fig. 1a). In line with these findings, cell cycle analysis demonstrated that Zyflamend alters cell cycle distribution in a dose-dependent manner. Indeed, Zyflamend treatment resulted in the enrichment of the G2/M fraction with 2 N DNA content, which was accompanied by a reduction in cell cycle progression through the G0/G1 and S phases (Fig. 1b-c). These results suggest that Zyflamend-induced inhibition of cell proliferation is mediated, at least in part, through cell cycle arrest in the G2/M phase.

In order to determine whether Zyflamend-induced inhibition of cell proliferation was associated with apoptotic cell death, we determined changes in apoptosis in β-TC6 cells treated with increasing doses of Zyflamend (0, 25, 50, 100, 200, 400, and 500 μg/ml) for 24 h using two approaches: the Guava Nexin Annexin V assay and Hoechst 33258 stain. Using the Annexin V assay, the percentages of both Annexin V positive/7-AAD negative cells (reflective of early apoptotic cells), and Annexin V positive/7-AAD positive cells (reflective of late apoptosis), exhibited a dose-dependent and significant increase in response to Zyflamend treatment (Fig. 1d-e). Consistent, with this observation, the number of Hoechst-positive cells was also higher in Zyflamend-treated cells compared to control cells (Fig. 1f-g). Hoechst 33258 is a nucleic acid dye that binds to condensed chromatin in the nucleus of apoptotic cells, thus giving an assessment of overall apoptotic cell death [34]. At a dose of 200, 400 and 500 μg/ml, the percentages of apoptotic cells were 30.44 ± 4.07%, 40.30 ± 1.30%, and 53.75 ± 4.45%, respectively, further emphasizing the pro-apoptotic effects of Zyflamend on these cells. Similar findings were obtained using the MTT assay (Fig. 1h).

### A human equivalent dose of Zyflamend induces apoptotic cell death in β-TC6 cells

**Fig. 2:**
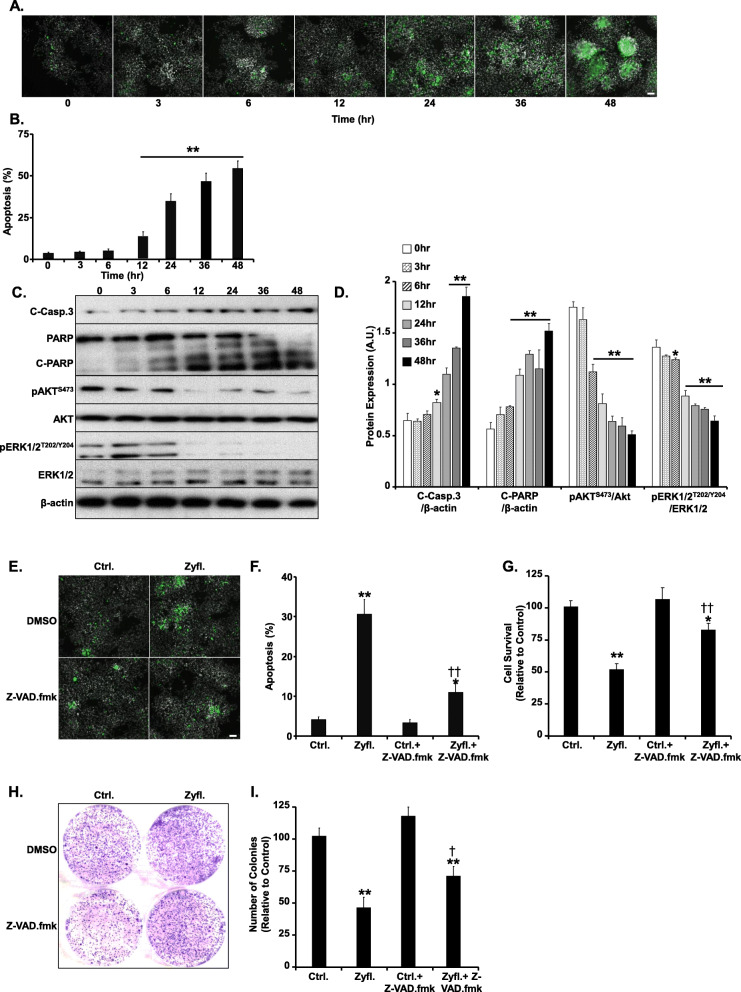
Zyflamend Induces Apoptotic Cell Death in β-TC6 Cells. **a-b** Effects of Zyflamend on chromatin condensation. Cells were treated with Zyflamend (200 μg/ml) for the indicated time and chromatin condensation was evaluated by fluorescence microscopy using Hoechst 33258. Representative images are shown. Scale bar: 50 μm. **b** Bar graphs represent the number of apoptotic cells (Hoechst positive) as means + SEM. **p < 0.01 indicates a significant difference between cells treated with Zyflamend and non-treated cells. **c-d** Immunoblots of key proteins in cell survival and apoptosis markers in cells treated with 200 μg/ml of Zyflamend for the indicated time. **d** Bar graphs represent cleaved caspase-3 (C-Casp. 3)/β-actin, cleaved PARP (C-PARP)/β-actin, pAKT/AKT, and pERK/ERK, as means + SEM. *p < 0.05, **p < 0.01 indicates a significant difference between cells treated with Zyflamend and non-treated cells. **e-f** Chromatin condensation in β-TC6 cells treated with 200 μg/ml Zyflamend with and without the pan-caspase inhibitor Z-VAD.fmk. Representative images are shown. Scale bar: 50 μm. **f** Bar graphs represent the number of apoptotic cells (Hoechst positive) as means + SEM. **g** Cell toxicity assay using the MTT method. Bar graphs represent the intensity of formazan staining reflective of the cell number and presented as means + SEM. **h-i** Colony formation assay. **i** Bar graphs represent the relative number of colonies in each condition determined by dividing the number of colonies for a given treatment by the total number of colonies in DMSO treated cells (Ctrl.) and expressed as a percentage. In **g** and **i**, *p < 0.05, **p < 0.01 indicate a significant difference between cells treated with Zyflamend and non-treated cells. †*p* < 0.05, ††*p* < 0.01 indicate a significant difference between cells treated with Z-VAD.fmk and non-treated

To further characterize the pro-apoptotic properties of Zyflamend, we conducted a time course analysis using a physiological relevant fixed dose of Zyflamend (200 μg/ml) [18]. This dose is representative of the maximum plasma concentration of a primary ingredient of Zyflamend (curcumin) that was reported in humans after oral administration [18]. At this dose, a marked increase in chromatin condensation and apoptotic cell number was observed after 24 h of treatment (Fig. 2a-b). Subsequently, markers of apoptosis and cell survival were investigated using Western blotting. Zyflamend induced cleavage of caspase-3 and its downstream target poly (ADP-ribose) polymerase (PARP) (Fig. 2c-d). In addition, we examined changes in the mitogen-activated protein kinases (MAP kinases) pathways in response to Zyflamend. Our data revealed that β-TC6 cells treated with Zyflamend exhibited a marked decrease in the phosphorylation of protein kinase B (AKT) and extracellular signal-regulated kinases (ERK), particularly after 12 h of treatment.

To determine whether Zyflamend-induced cell death was associated with the caspase dependent pathways of apoptosis, we tested whether blocking caspases’ activity using carbobenzoxy-valyl-alanyl-aspartyl-[O-methyl]-fluoromethylketone (Z-VAD.fmk) could inhibit Zyflamend-induced chromatin condensation and apoptosis. Z-VAD.fmk is a potent cell permeable pan-caspase inhibitor, which acts by irreversibly binding to the catalytic site of the caspase proteases, and thus inhibiting their activities. Our study shows that pre-treatment with Z-VAD.fmk caused a significant decreased in the levels of chromatin condensation in Zyflamend-treated cells (Fig. 2e-f). Additionally, Z-VAD.fmk treatment alleviated Zyflamend-induced cell toxicity as judged by the MTT (Fig. 2**g**) and the colony formation (Fig. 2h-i) assays. Taken together, our findings indicate that Zyflamend treatment reduces cell viability and induces cell death through the induction of the apoptotic machinery in β-TC6 cells.

### Zyflamend induces ER stress, apoptosis and autophagy responses in β-TC6 cells

**Fig. 3:**
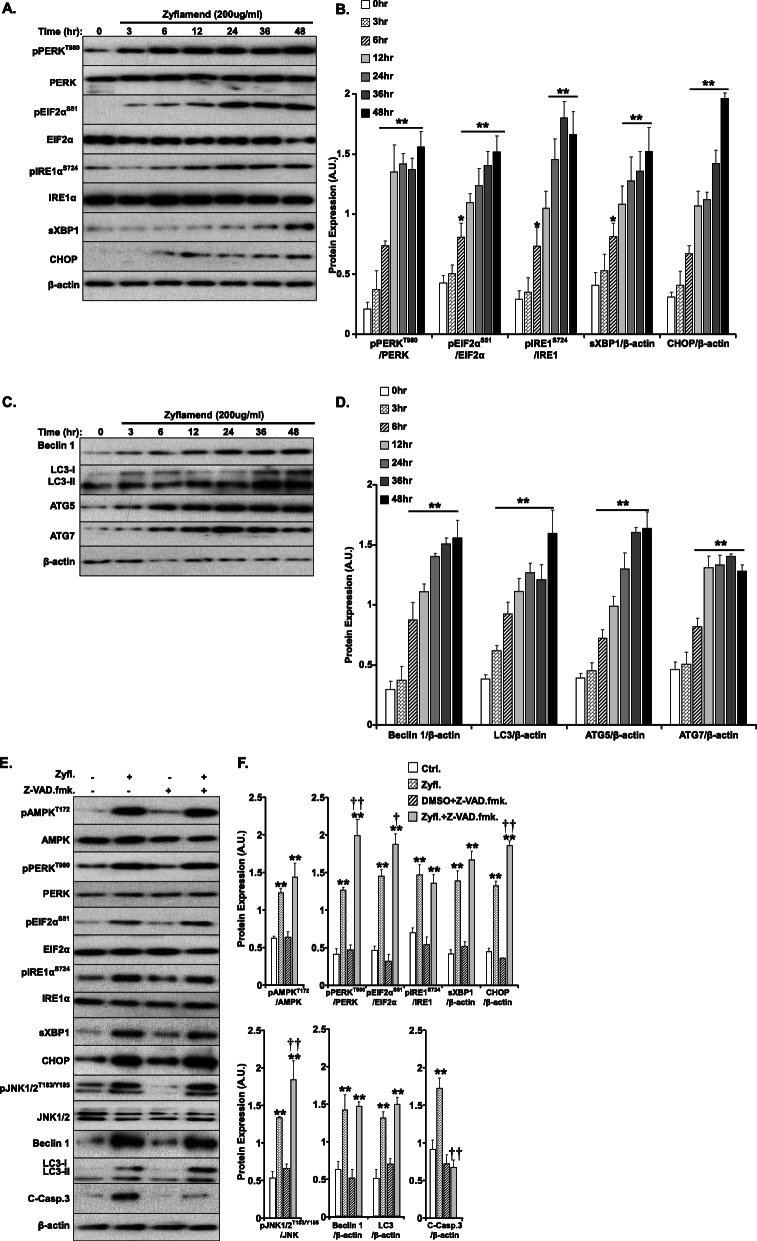
Zyflamend Induces Inflammatory, ER Stress, and Autophagy Responses in β-TC6 Cells. **a-b** Total cell lysates from control and Zyflamend treated cells for 3, 6, 12, 24, 36 and 48 h were immunoblotted for ER stress markers: pPERK, pEIF2α, pIRE1α, their respective unphosphorylated proteins, sXBP1, CHOP, and β-actin as a loading control. Representative immunoblots are shown. **b** Bar graphs represent pPERK/PERK, pEIF2α/EIF2α, pIRE1/IRE1, sXBP1/β-actin, and CHOP/β-actin as means + SEM. *p < 0.05, **p < 0.01 indicate a significant difference between cells treated with Zyflamend and non-treated cells. **c-d** Markers of autophagy were examined in the same lysates using antibodies against Beclin 1, LC3, I&II, ATG5, ATG7, and β-actin as a loading control. **d** Bar graphs represent Beclin 1/β-actin, LC3/β-actin, ATG5/β-actin, and ATG7/β-actin as means + SEM. *p < 0.05, **p < 0.01 indicate a significant difference between cells treated with Zyflamend and non-treated cells. **e-f** Immunoblots of key proteins in autophagy, AMPK, ER stress, and apoptosis signaling in β-TC6 cells treated with 200 μg/ml Zyflamend with and without the pan-caspase inhibitor Z-VAD.fmk. Representative immunoblots are shown. **f** Bar graphs represent pAMPK/AMPK, pPERK/PERK, pEIF2α/EIF2α, pIRE1α/IRE1α, sXBP1/β-actin, CHOP/β-actin, pJNK/JNK, Beclin 1/β-actin, LC3I&II/β-actin, and cleaved caspase3/β-actin as means + SEM. *p < 0.05, **p < 0.01 indicate a significant difference between cells treated with Zyflamend and non-treated cells. †*p* < 0.05, ††*p* < 0.01 indicate a significant difference between cells treated with Z-VAD.fmk and non-treated cells

A plethora of intrinsic and extrinsic pathways can lead to apoptosis in response to stressors, including ER stress, and autophagy, among many more. Therefore, in order to dissect the precise molecular mechanism mediating the pro-apoptotic effects of Zyflamend, we examined the activation of key signaling molecules related to these pathways. Zyflamend (200 μg/ml) significantly induced ER stress as judged by the activation of ER transmembrane sensors, protein kinase RNA-like endoplasmic reticulum kinase (PERK) and inositol-requiring transmembrane kinase/endoribonuclease 1α (IRE1α), along with downstream targets such as eukaryotic translation initiation factor 2 alpha (EIF2α) and C/EBP homologous protein (CHOP) by Western blotting. Zyflamend induced ER stress as evidenced by increased PERK (Thr^980^), EIF2α (Ser^51^) and IRE1α (Ser^724^) phosphorylation (Fig. 3a). Furthermore, the level of CHOP expression was elevated, a direct downstream target of both the PERK and IRE1 pathways. The activation of CHOP, a potent inducer of apoptotic cell death [35, 36], in response to ER stress (Fig. 3a-b), strengthens our conclusions of Zyflamend-induced apoptosis in these cells. Moreover, Zyflamend has been shown to activate AMPK, and our results recapitulate these previous findings [20].

The AMPK signaling pathway has been shown to regulate autophagy and cell death [37]. Therefore, in order to assess whether Zyflamend induces autophagy in β-TC6 cells, we immunoblotted for autophagy-related proteins. We observed a time dependent increase in beclin 1, microtubule-associated proteins 1A/1B light chain (LC3-I & II), and autophagy-related proteins 5 and 7 (ATG5/7) (Fig. 3c-d). The increase in the expression of these proteins is indicative of elevated autophagy in these cells. Because ER stress, inflammation, and autophagy can all lead to apoptosis, we used the pan-caspase inhibitor Z-VAD.fmk to determine which pathway might be responsible for the pro-apoptotic effects of Zyflamend. Our data shows that, while there was a significant attenuation of Zyflamend-induced cleavage of caspase-3 and its downstream target PARP, treatment with Z-VAD.fmk had no effects on Zyflamend-induced activation of the AMPK, autophagy, and ER stress signaling cascades (Fig. 3e-f). These findings suggest that ER stress, autophagy, and MAP kinases pathways are upstream of the apoptotic signaling cascade that might be mediating the pro-apoptotic effects of Zyflamend in β-TC6 cells.

### Zyflamend-induced cell death is mediated through the ER stress/JNK/autophagy pathway

**Fig. 4:**
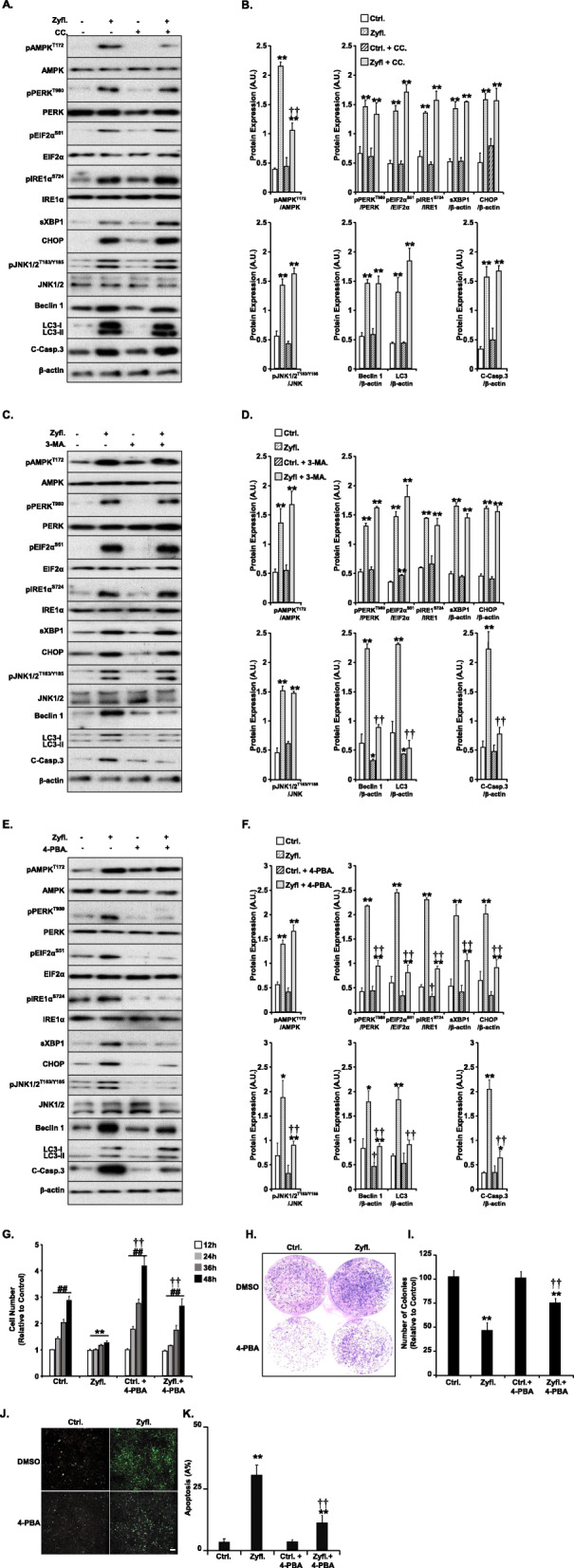
Zyflamend-Induced Cell Death is Mediated through the ER Stress/Autophagy Pathways. Total cell lysates from control cells treated with Zyflamend and non-treated cells with or without AMPK inhibitor (Compound C: CC), (**a**-**b**), autophagy inhibitor (3-MA) (**c**-**d**), or ER stress inhibitor (4-PBA; **e**-**h**) were immunoblotted for markers of inflammation, ER stress, autophagy, and cell death. Cells were pre-treated with the indicated inhibitors 12 h prior to adding Zyflamend or DMSO (as a vehicle control) for an additional 24 h. In **b**, **d**, and **f** Bar graphs represent) Bar graphs represent pAMPK/AMPK, pPERK/PERK, pEIF2α/EIF2α, pIRE1α /IRE1α, sXBP1/β-actin, CHOP/β-actin, pJNK/JNK, Beclin 1/β-actin, LC3II&II/β-actin, and cleaved caspase3/β-actin as means + SEM. *p < 0.05, **p < 0.01 indicate a significant difference between cells treated with Zyflamend and non-treated cells. †*p* < 0.05, ††*p* < 0.01 indicate a significant difference between cells treated with the indicated inhibitor and non-treated cells (CC, 3-MA, or 4-PBA). **g** Cells were treated with 200 μg/ml of Zyflamend for the indicated time with or without 4-PBA. Bar graphs represent the intensity of SRB staining reflective of the cell number and presented as means + SEM from at least three independent experiments. *p < 0.05, **p < 0.01 indicate a significant difference between cells treated with Zyflamend and non-treated cells. †*p* < 0.05, ††*p* < 0.01 indicate a significant difference between non-treated and cells treated with 4-PBA. **h-i** Colony formation assay. **i** Bar graphs represent the relative number of colonies in each condition determined by dividing the number of colonies for a given treatment by the total number of colonies in DMSO treated cells (Ctrl.) and expressed as a percentage. **j-k** Chromatin Condensation in cells treated with 200 μg/ml of Zyflamend alone or in combination with 4-PBA for 24 h. Representative images are shown. Scale bar: 50 μm. **i** Bar graphs represent the number of apoptotic cells (Hoechst positive) as means + SEM from at least three independent experiments. In **i** and **k**, **p < 0.01 indicates a significant difference between cells treated with Zyflamend and non-treated cells. ††*p* < 0.01 indicates a significant difference between cells treated with 4-PBA and non-treated cells

The exact molecular mechanism(s) leading to apoptosis by Zyflamend in cancer cells has/have not been revealed yet, although recent studies have supported the role of AMPK in the regulation of cancer cell growth, bioenergetics, autophagy, and cell death. To investigate the potential role of AMPK in Zyflamend-induced apoptosis, we pre-treated cells with the AMPK inhibitor compound C (CC; 5 μM) for 12 h prior to Zyflamend treatment for an additional 24 h. The dose and duration of exposure were determined based on the ability of compound C to reverse AMPK-dependent inhibitory phosphorylation of acetyl-CoA carboxylase (ACC) (data not shown). Cells were then examined for AMPK activation as well as activation of inflammation, ER stress, autophagy, and cell death **(**Fig. 4a**)**. While the level of phosphorylated AMPK was reduced in co-treated cells, pre-treatment with compound C had no effects on Zyflamend-induced JNK phosphorylation, ER stress, autophagy, or cell death (Fig. 4a-b). These data suggest that Zyflamend-induced apoptosis in β-TC6 cells is independent of AMPK activation. Next, we sought to examine whether blocking autophagy using 3-MA could protect cells against Zyflamend-induced apoptosis. 3-MA inhibits autophagy by blocking autophagosome formation via the inhibition of class I and class III phosphatidylinositol 3-kinases (PI3K) [38]. Cells were pre-incubated with 3-MA (5 nM) for 12 h prior to Zyflamend treatment. Co-treatment with Zyflamend and 3-MA significantly decreased the expression of beclin 1, LC3, and cleaved caspase-3, but had no effects on ER stress markers nor on AMPK phosphorylation (Fig. 4c-d). These data suggest that autophagy mediates Zyflamend-induced apoptosis and that both AMPK and ER stress activation by Zyflamend occur upstream of autophagy and apoptosis.

The relationship between these two fundamental processes, ER stress and autophagy, is complex and poorly understood. Recent literature demonstrates that both pathways display dual roles in cell survival in multiple cancer cell lines. Similar to ER stress, autophagy has been shown to promote cell survival by clearing unwanted components from the cells. Nonetheless, a considerable body of evidence also indicates that both autophagy and ER stress can lead to apoptosis in tumor cells. In addition to this, a growing body of literature supports existing crosstalk between the two pathways [39, 40]. However, which pathway is upstream of the other is yet to be determined. Our data suggest that Zyflamend-induced autophagy is likely to be downstream of ER stress. To test this hypothesis, we pre-treated β-TC6 cells with an ER stress inhibitor 4-phenylbutyrate (4-PBA; 5 mM for 12 h) prior to Zyflamend treatment, and we examined changes in inflammation, ER stress, autophagy, and cell death (Fig. 4e-f). 4-PBA is a cell permeant chemical chaperone that has been shown to inhibit ER stress and ER stress-induced apoptosis in many cancer cell types, including pancreatic cancer cells [41, 42]. Our findings show a profound decrease in ER stress, autophagy, and cell death markers in response to Zyflamend (Fig. 4e-f) when cells were pre-treated with 4-PBA. Additionally, 4-PBA treatment alleviated the decrease in cell proliferation (Fig. 4g) and colony formation (Fig. 4h-i) caused by Zyflamend. Furthermore, pre-treatment of β-TC6 cells with 4-PBA reduced Zyflamend-induced chromatin condensation (Fig. 4j-k). Conversely, 4-PBA did not alter the activation of AMPK by Zyflamend (Fig. 4e-f), suggesting that ER stress occurs upstream of autophagy and apoptotic cell death.

**Fig. 5:**
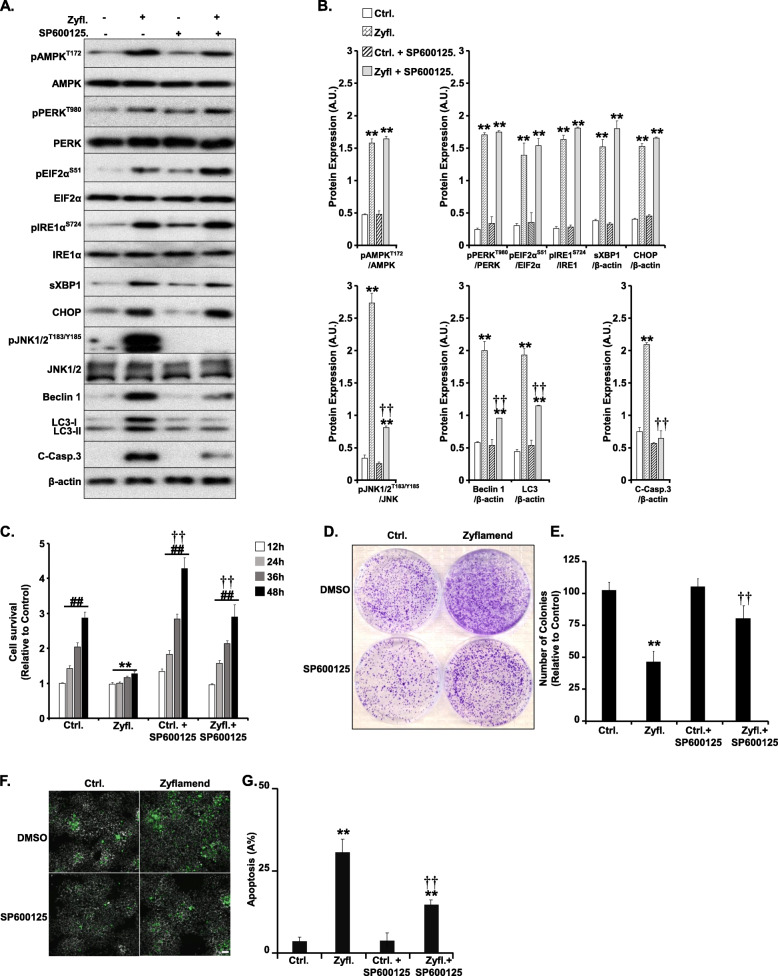
JNK mediates Zyflamend-Induced Cell Death. **a**, **b** Cells were treated with 200 μg/ml of Zyflamend for the indicated time with or without SP600125. Total cell lysates were immunoblotted for markers of inflammation, ER stress, autophagy, and cell death. Bar graphs represent pAMPK/AMPK, pPERK/PERK, pEIF2α/EIF2α, pIRE1α /IRE1α, sXBP1/β-actin, CHOP/β-actin, pJNK/JNK, Beclin 1/β-actin, LC3II&II/β-actin, and cleaved caspase3/β-actin as means + SEM. *p < 0.05, **p < 0.01 indicate a significant difference between cells treated with Zyflamend and non-treated cells. †*p* < 0.05, ††*p* < 0.01 indicate a significant difference between cells treated with SP600125 and non-treated cells. **c** Bar graphs represent the intensity of SRB staining reflective of the cell number and presented as means + SEM from at least three independent experiments. *p < 0.05, **p < 0.01 indicate a significant difference between cells treated with Zyflamend and non-treated cells. †*p* < 0.05, ††*p* < 0.01 indicate a significant difference between cells treated with SP600125 and non-treated cells. **d-e** Colony formation assay. **e** Bar graphs represent the relative number of colonies in each condition determined by dividing the number of colonies for a given treatment by the total number of colonies in DMSO treated cells (Ctrl.) and expressed as a percentage. **f-g** Chromatin condensation in cells treated with 200 μg/ml of Zyflamend alone or in combination with SP600125 for 24 h . Representative images are shown. Scale bar: 50 μm. **g** Bar graphs represent the number of apoptotic cells (Hoechst positive) as means + SEM from at least three independent experiments. In **E** and **G**, **p < 0.01 indicates a significant difference between cells treated with Zyflamend and non-treated cells. ††*p* < 0.01 indicates a significant difference between non-treated and cells treated with SP600125

Previous studies have shown that the ER stress sensor IRE1 may promote autophagy through the TRAF2/ASK1/JNK pathway [43, 44]. To test this hypothesis, we treated β-TC6 cells with SP600125 (a selective JNK inhibitor) and investigated changes in inflammation, ER stress and proliferation in response to Zyflamend treatment (Fig. 5). As expected, cells pre-treated with SP600125 exhibited a significant reduction in the phosphorylation of JNK and reduced expression of autophagy and cell death markers in response to Zyflamend (Fig. 5a-b). Likewise, JNK inhibition protected β-TC6 cells from Zyflamend-induced reduction in cell survival (Fig. 5c), colony formation (Fig. 5d-e), and chromatin condensation (Fig. 5f-g). However, pre-treatment with SP600125 did not alter Zyflamend-induced activation of the AMPK and ER stress pathways (Fig. 5a-b). Altogether, the above findings suggest that Zyflamend is capable of reducing survival and inducing apoptosis in the β-TC6 murine insulinoma cells through a complex mechanism involving ER stress-mediated JNK activation, which in turn induces autophagy followed by the induction of apoptosis.

### Zyflamend enhances the effects of streptozotocin and adriamycin

**Fig. 6:**
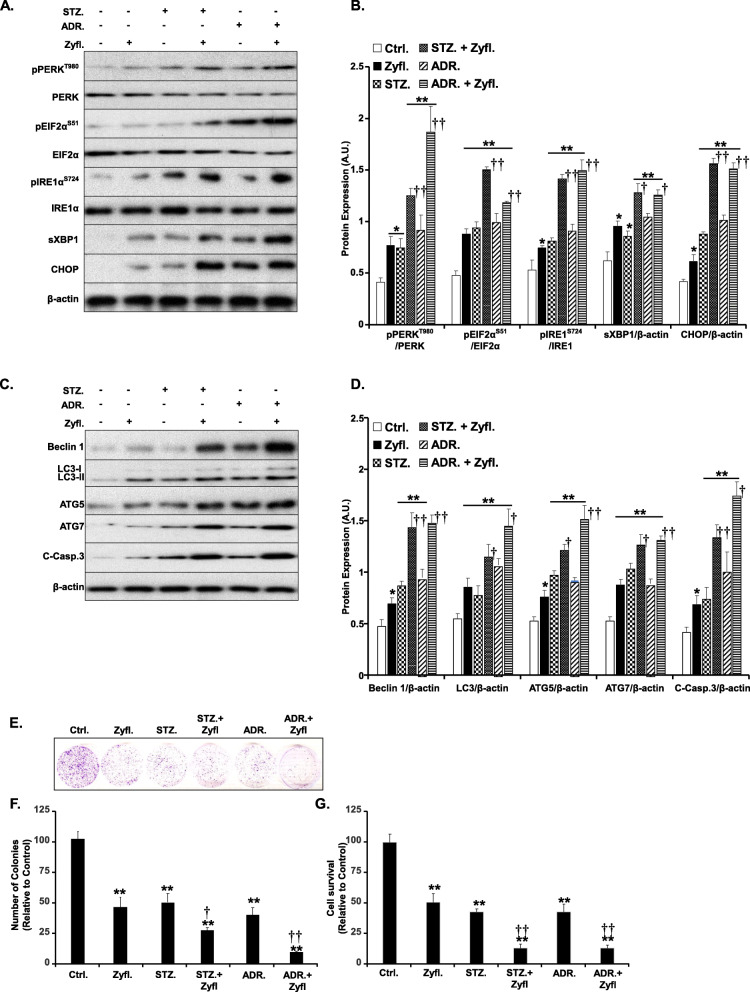
Zyflamend Exacerbates the Effects of Streptozotocin and Adriamycin on ER Stress, Autophagy, and Cell Death in β-TC6 Cells. Total cell lysates from control cells treated with Zyflamend and non-treated cells with or without streptozotocin (STZ; 5 mM) or adriamycin (ADR; 5 μM) for 24 h were immunoblotted for markers of ER stress **(a-b)**, autophagy, and cell death **(c-d)**. Representative immunoblots are shown. **b** Bar graphs represent pPERK/PERK, pEIF2α/EIF2α, pIRE1/IRE1, sXBP1/β-actin, and CHOP/β-actin as means + SEM. **d** Bar graphs represent Beclin 1/β-actin, LC3/β-actin, ATG5/β-actin, ATG7/β-actin, and cleaved caspase-3 / β-actin as means + SEM. **e-f** Colony formation assay. **f** Bar graphs represent the relative number of colonies in each condition determined by dividing the number of colonies for a given treatment by the total number of colonies in DMSO treated cells (Ctrl.) and expressed as a percentage. **g** Cell toxicity assay using the MTT method. Bar graphs represent the intensity of formazan staining reflective of the cell number and presented as means + SEM. In **b, d, f** and **g**, *p < 0.05, **p < 0.01 indicate a significant difference between cells treated with Zyflamend and non-treated cells. †*p* < 0.05, ††*p* < 0.01 indicate a significant difference between cells treated with Zyflamend combined with the chemotherapeutic agents (streptozotocin or adriamycin) and cells treated with streptozotocin or adriamycin only

It was our objective to assess the effectiveness of combining Zyflamend with chemotherapeutics commonly used to treat endocrine pancreatic cancer. Thus, we examined the effects of Zyflamend treatment alone or in combination with streptozotocin (STZ) or adriamycin (ADR) for 24 h. Total cell lysates were then immunoblotted for various markers of ER stress, autophagy, and cell death. Both STZ and ADR caused a significant increase in ER stress as demonstrated by the increased phosphorylation of both PERK and its downstream target EIF2α, along with increased phosphorylation of IRE1 and the splicing of its downstream target X-Box Binding Protein 1 (XBP1). Likewise, the expression of CHOP was significantly higher in cells treated with Zyflamend, streptozotocin, or adriamycin. Zyflamend treatment exacerbated the effects of streptozotocin and adriamycin on ER stress (Fig. 6a-b). Additionally, Zyflamend enhanced STZ and ADR-induced expression of autophagy (beclin 1, LC3-I & II, ATG5, and ATG7) and apoptosis (cleaved caspase-3) markers (Fig. 6c-d). Consistent with these findings, Zyflamend exacerbated STZ and ADR-induced cell toxicity as demonstrated by the colony formation (Fig. 6e-f) and the MTT (Fig. 6g) assays. Notably, these findings were corroborated in rat pancreatic insulinoma RIN-5F cells with a combination of Zyflamend and STZ or ADR (Supplementary Fig. S1). Taken together, our study revealed that Zyflamend may serve as an adjuvant capable of enhancing the effectiveness of standard cancer therapies.

## Discussion

Pancreatic cancer is one of the most untreatable forms of malignancy. The abysmal survival rates for pancreatic cancer diagnosis, leave patients seeking alternatives. The success of current therapies has been limited, and in many cases, ineffective. In addition, these therapies often rely on invasive procedures and high doses that can lead to cytotoxicity and collateral side effects. The need for innovative solutions to these problems has never been higher, and the search for more effective therapies persists. Plant-based compounds are widely considered a promising avenue for combined and synergistic enhancement of known therapies [45, 46].

Emerging evidence supports the anti-tumor and pro-apoptotic effects of Zyflamend on various types of cancers such as prostate [13], melanoma [14], and oral cancer [15]. In this study, we uncover novel insight into the molecular mechanisms through which Zyflamend could prove beneficial both alone and as an adjunct therapy in targeting pancreatic cancer. We demonstrate that Zyflamend is capable of inducing cell death and reducing cell survival in a time-and dose-dependent manner. Furthermore, we demonstrate that Zyflamend induces ER stress, which in turn activates the JNK pathway leading to autophagy and caspase-mediated cell death (Schema 1). Additionally, Zyflamend enhances the effects of streptozotocin and adriamycin on cell death and survival, two chemotherapeutic compounds used to treat PNETs. Taken together, these findings promote Zyflamend as a potential adjuvant to PNETs therapy.

**Schema 1:**
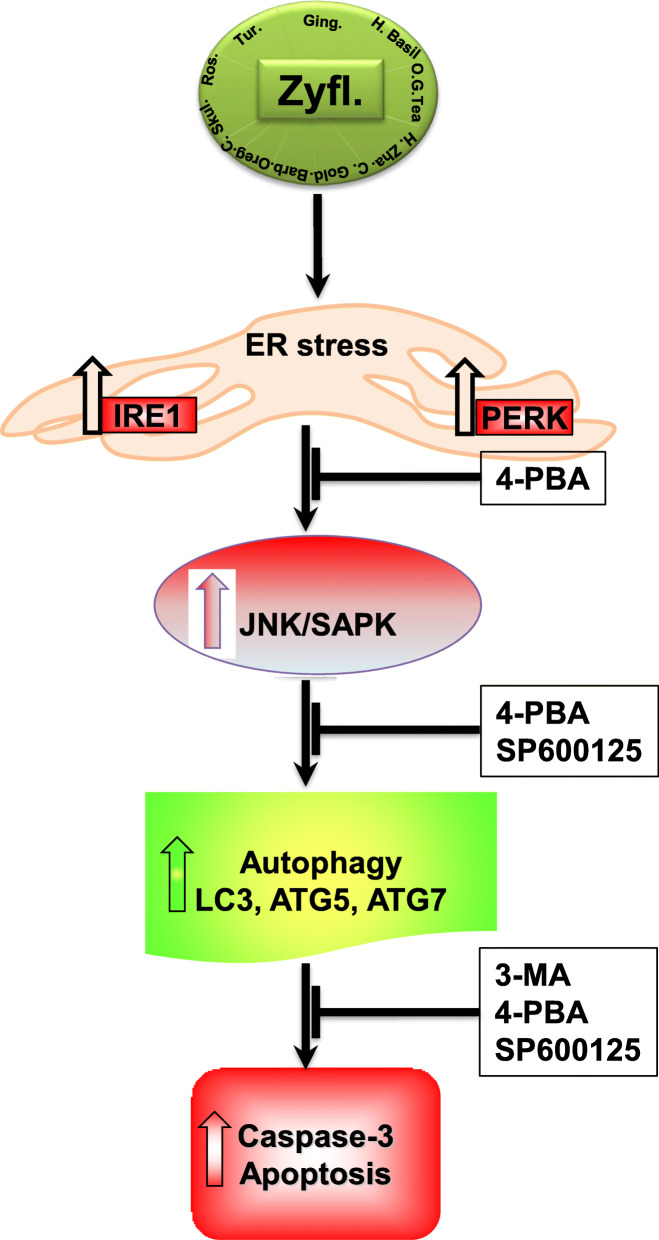
Overview of the Pro-Apoptotic Effects of Zyflamend in Pancreatic Cancer Cells. Zyflamend treatment induced ER stress, autophagy, MAP kinases and apoptosis in pancreatic β-TC6 Cells. Previous studies have shown that activation of AMPK in pancreatic cancer cells by chemotherapeutic agens induces apoptosis and autophagy via the AMPK/mTOR signaling pathway (dotted lines) [47]. Similarly, activation of the JNK/SAPK pathway [48] by stressors and chemotherapeutic agents can lead to apoptotic cell death of pancreatic cancer cells. Our study shows that treatment with pan-caspase inhibitor Z-VAD.fmk prevented the cleavage of caspase 3 but had no effects on AMPK, autophagy, or ER stress. Zyflamend treatment activated AMPK; however, treatment with AMPK inhibitor CC did not result in changes to ER stress, apoptosis, autophagy, or JNK signaling pathways. Autophagy inhibition with 3-MA inhibited autophagy and apoptosis only. Zyflamend activated both the IRE-1 and PERK branches of the ER stress mediated UPR and resulted in the subsequent upregulation of the downstream JNK signaling pathway. ER stress inhibition with 4-PBA resulted in decreased JNK activation, autophagy, and apoptosis. While inhibition of JNK with SP600125 significantly reduced the the level of both, apoptosis and autophagy

Our findings are in line with Zyflamend cancer research across a broad spectrum of experimental platforms [13–15, 21, 22, 24]. Research has revealed that the individual extracts and constituents contained within Zyflamend could possess therapeutic potential and value as backed by a large number of investigations. For instance, curcumin, a polyphenol found in turmeric, has shown promise in a wide variety of cancer research [17, 49]. Curcumin could inhibit carcinogenesis through a variety of proposed pathways and processes including, the inhibition of angiogenesis and tumorigenesis [50], the disruption of the mTOR-raptor complex and subsequently decreased phosphorylation and activation of AKT/mTOR signaling cascade [51, 52]. In addition, curcumin can lead to the inactivation of the PI3K/AKT pathway and subsequently induce apoptosis through increased mitochondrial P53 and BCL2-associated X protein (BAX) translocation [53]. Furthermore, curcumin can also regulate autophagy turnover in an AMPK/ULK1 dependent manner [49]. Moreover, curcumin can induce cell cycle arrest and apoptosis supporting its potential as an adjuvant therapy through increasing the sensitivity of multidrug resistant pancreatic cancer tumors to gemcitabine treatment [54].

Likewise, ginger (another component of Zyflamend) and its derivatives have been evaluated for their anti-cancer properties [55, 56] and were shown to modulate major signaling pathways important for cancer survival and proliferation in several cancer models. Indeed, ginger and its derivatives were demonstrated to induce cancer cell death through modulation of the NF-*κ*B, PI3K/AKT/mTOR, inflammation, cell cycle, angiogenesis, and apoptotic signaling pathways [57, 58]. In pancreatic cancer research, ginger and its major pungent components (gingerol, shogaol, and zerumbone) were reportedly shown to downregulate and/or inhibit NF-*κ*B signaling across a variety of pancreatic cancer cell lines [58, 59]. For example, 6-shogaol was demonstrated to sensitize PANC-1 cells to gemcitabine through inhibiting NF-*κ*B, cyclin D1, Bcl-2, COX-2, resulting in increased apoptosis [60]. While a majority of the studies have examined the effects of phytochemicals on PDAC, investigations targeting their effects on PNETs remains limited. For instance, isolated polyphenols of rosemary, such as carnosic acid and rosmarinic acid, were shown to cause an increase in TNFα and nitrate levels leading to the induction of apoptosis in rat insulinoma (RINm5F) cells [61, 62]. Supportively, treatment of pancreatic endocrine HP62 cells with the phytochemical Devil’s club *Oplopanax horridus* (DC), a close relative of the ginseng family, induced ER stress and apoptosis and exacerbated the anti-proliferative effects of gemcitabine, cisplatin, and paclitaxel [63]. Likewise, carnosic acid RE derivatives also exhibited tumor suppressive potential in a PANC-1 model of PDAC [64]. Careful consideration of the overall biological implications of natural compounds targeting PDAC and PNETs may reveal critical insight into pancreatic cancer oncogenesis, allowing for therapeutic enhancement.

Human PDAC cells treated with 6-gingerol exhibited a cell cycle arrest at the G1 phase through decreased cyclin-dependent kinase (CDK) expression and reduced phosphorylation of retinoblastoma protein (pRb) [65]. Similarly, zerumbone, another isolated component of ginger, demonstrated pro-apoptotic effects on PANC-1 cells through the upregulation of p21, p53, and increased ROS production [66]. Furthermore, rosemary and its constituents have proven effective in a wide variety of cancer research models [61, 67] through several mechanisms. Petiwala and colleagues demonstrated that rosemary extracts activate the ER stress response and induced apoptosis in 22Rv1 and LNCaP prostate cancer cells. In these cell lines, the rosemary extracts also increased the expression of BAX, cleaved caspase-3, CHOP, and IRE1α, in a mechanism similar to our findings [68] Finally, the Zyflamend component holy basil has also shown promise in pancreatic cancer research as it inhibited tumorigenesis in both murine and in vitro models and promoted apoptosis [69]. Taken together, these findings demonstrate the potential for combining these herbal extracts to target pancreatic cancer.

The development and progression of pancreatic cancer have been linked to the activation and inhibition of a variety of cell signaling pathways. In this study, we explored the role of Zyflamend on cell survival, cell cycle, and cell death. We demonstrate that Zyflamend attenuates cell survival, causes G2/M cell cycle arrest and promote apoptotic cell death in a dose and time dependent manner (Schema 1). While 800 μg/ml completely inhibited cell growth, 200 μg/ml was sufficient to reduce cell survival significantly. Based on previously described findings, we chose 200 μg/ml for further exploration because this dose represents the maximum plasma concentration of one of Zyflamend’s constituents, curcumin, in humans following supplementation [18]. Importantly, previous studies have revealed that bioactive compounds such as those found in Zyflamend, can be more effective when combined. Notably, compounds exhibiting relatively low bioactivity in isolation often show increased activation when combined with another bioactive originating from the same source [18]. Moreover, Zyflamend doses at or below 200 μg/ml have proven effective, to varying degrees, in either inducing apoptosis and/or inhibiting proliferation of several tumor-derived cell lines, including pancreatic [22] and prostate [70] cancer cells. In agreement with these studies, we report here that this dose was sufficient to significantly induce apoptotic cell death in pancreatic cancer cells. On the other hand, while the individual components of Zyflamend show potential in targeting cancer, the doses often required to elicit responses exceed physiological relevance. For instance, curcumin dose required to elicit the inhibition of AKT phosphorylation occurred at high doses (> 40 μM) in a previously mentioned study [51]. In addition, the dose required to generate an anti-tumor response to a polyphenolic extract from ginger, 6-shogaol, was relatively high, both in vitro (15–20 uM) and in vivo (50 mg/Kg) in a pancreatic cancer model [60]. Research on rosemary extracts shows significant variation in the doses used in cancer research with in vivo doses reported from a low of 100 mg/Kg/day to as high as 3333.3 mg/Kg/day [61]. Furthermore, a dose of 6 g of green tea per day given to prostate cancer patients led to varying degrees of collateral cytotoxicity [71]. In order to develop translationally valid phytochemical research, it is essential to carefully consider several factors including the human equivalent dose, the clinical validity of the experimental model, and the molecular mechanisms involved.

In order to determine the molecular mechanisms mediating Zyflamend’s actions, we explored pathways that are critical for pancreatic cancer cell survival and proliferation. We found that Zyflamend induced the cleavage of caspase-3 along with its downstream target PARP. This finding is in alignment with other studies showing similar activation of the apoptotic cascade in response to Zyflamend treatment in colon, prostate, pancreatic (Panc-28) and bladder cancer models [23, 25]. Next, we probed for changes in the MAPK pathways in response to Zyflamend. As aforementioned, the MAPK pathways include MEK1/ERK1/2/p90 RSK, P38 MAPK, and JNK/c-JUN pathways. In conjunction with the PI3K/AKT/mTOR pathway, these proteins and pathways are responsible for key physiological responses such as cell survival, protein synthesis, cell proliferation, growth, migration, and apoptosis in both normal and cancerous cells [72, 73]. In addition, elevated activation of the PI3K signaling cascade is observed in a majority of cancers. Therefore, targeting the PI3K pathway with natural compounds could provide invaluable therapeutic potential to cancer patients. At much higher doses, individual components found in Zyflamend such as curcumin, ginger (6-shogaol) [74], and rosemary [16] have all been shown to inhibit the phosphorylation of AKT and cell survival of various cancer types. In addition, curcumin has been shown to inhibit the phosphorylation of AKT in a pancreatic cancer cell line (PANC-1) [75]. In line with these findings, our data demonstrate that Zyflamend treatment caused a significant reduction in both ERK1/2 and AKT phosphorylation.

A key finding from this research is that, in addition to inducing autophagy, Zyflamend also upregulates ER stress and through this could reduce the survival of pancreatic cancer cells. The ER stress response is a set of highly regulated mechanisms sensitive to a wide variety of physiological and pathological conditions that affect protein synthesis and folding. One of the mechanisms through which misfolded and/or unfolded proteins are handled by the ER is through ER-associated protein degradation (ERAD). As improperly folded aggregated proteins reach excessive thresholds, the primary functions of the ER stress response can be overwhelmed. To cope with this rising stress level, the autophagy-lysosome dependent ERAD (type II) machinery can be activated [76, 77]. If the level of unfolded proteins continues to rise, the stress can overwhelm both autophagy and the ER’s capacity to function, leading to the induction of programmed cell death [77]. In the environment of tumorigenesis, the elevated levels of metabolic stress can often lead to the activation of autophagy and ER stress responses [78, 79]. The findings of Klieser et al., in BON1 cells, support the potential in targeting ER stress as a therapeutic approach to PNETs [80]. However, the activation of autophagy and the proper functioning of ER stress responses act in duality where properly functioning can protect cancer from the harsh microenvironment, and when critical thresholds are reached, it can initiate cell death [79]. In this study, we demonstrated that Zyflamend treatment led to the activation of two branches of the ER stress mediated unfolded protein response (UPR) through increased phosphorylation of PERK and IRE1α and activation of their downstream targets EIF2a and sXBP1. In addition, this was accompanied by elevated levels of CHOP, an ER stress mediated apoptosis inducer. Taken together, our findings show that Zyflamend can act as an inducer of apoptotic cell death through modulation of ER stress and autophagy and support the hypothesis of the potential beneficial effect of targeting ER stress and autophagy for the treatment of PNETs. In recent times, both natural and synthetic compounds have been explored regarding ER stress and autophagy mediated apoptosis in various cancer models [81]. However, this study is the first to delineate how natural compounds can act synergistically in the coordination of ER stress, MAP kinases, and autophagy signaling to induce cell death in pancreatic cancer cells.

## Conclusions

In summary, our study shows that, through a unique combination of 10 herbs, the therapeutic potential for targeting PNETs (and possibly other types of cancer) could be enhanced in an additive or synergistic manner. It is our hope, the findings from this study may pave the way towards pre-clinical validation of the beneficial effects of Zyflamend as a potential adjuvant for the treatment for PNETs. It is also our hope that finding from this study could be soon translated into clinical trials aimed at developing safer and more potent therapeutic approaches capable of improving pancreatic cancer patient outcomes and quality of life.

## Supplementary information

**Additional file 1: Supplementary Table 1.** List of Primary Antibodies and Conditions of Use. **Supplementary Table 2.** Composition of Zyflamend [82].

**Additional file 2: Supplementary Fig. 1.** Zyflamend Causes Toxicity and Exacerbates the Effects of Streptozotocin and Adriamycin on ER Stress, Autophagy, and Cell Death in RIN-5F Cells. **A-B)** Colony formation assay. **B)** Bar graphs represent the relative number of colonies in each condition determined by dividing the number of colonies for a given treatment by the total number of colonies in DMSO treated cells (Ctrl.) and expressed as a percentage. **C)** Cell toxicity assay using the MTT method. Bar graphs represent the intensity of formazan staining reflective of the cell number and presented as means + SEM. **D-G**) Total cell lysates from control cells treated with Zyflamend and non-treated cells with or without streptozotocin (STZ; 5 mM) or adriamycin (ADR; 5 μM) for 24 h were immunoblotted for markers of ER stress **(D-E)**, autophagy, and cell death **(F-G)**. Representative immunoblots are shown. **E)** Bar graphs represent pPERK/PERK, pEIF2α/EIF2α, pIRE1/IRE1, sXBP1/β-actin, and CHOP/β-actin as means + SEM. *p < 0.05, **p < 0.01 indicate a significant difference between cells treated with Zyflamend and non-treated cells. **G**) Bar graphs represent Beclin 1/β-actin, LC3/β-actin, ATG5/β-actin, ATG7/β-actin, and cleaved caspase-3 / β-actin as means + SEM. In **C, E,** and **G** *p < 0.05, **p < 0.01 indicate a significant difference between cells treated with Zyflamend and non-treated cells. †*p* < 0.05, ††*p* < 0.01 indicate a significant difference between cells treated with Zyflamend combined with the chemotherapeutic agents (streptozotocin or adriamycin) and cells treated with streptozotocin or adriamycin only.

## Data Availability

All data generated or analyzed during this study are included in this published article.

## Abbreviations

ADR: Adryamicin
7-AAD: 7-amino-actinomycin D
ACC: Acetyl-CoA carboxylase
AKT: Protein kinase B
AMPK: AMP-activated protein kinase
ATF-4: Activating transcription factor 4
ATG: Autophagy related protein
BAX: Bcl2-associated X protein
β-TC6: Beta-TC-6 pancreatic insulinoma cells
CC: Compound C
Cdk: Cyclin-dependent kinase
CHOP: C/EBP homologous protein
COX: Cyclooxygenase
DMSO: Dimethyl-sulfoxide
DTT: Dithiothreitol
EGCG: Epigallocatechin gallate
EGTA: Ethylene glycol-bis-(2-aminoethyl)-N,N,N′,N′-tetraacetic acid
EIF2α: Eukaryotic translation initiation factor 2 alpha
ER: Endoplasmic reticulum
ERAD: ER-associated degradation
ERK: Extracellular signal–regulated kinases
FBS: Fetal bovine serum
FITC: Annexin V- Fluorescein isothiocyanate
GPR78: G protein-coupled receptor
HSP: Heat shock protein
Huh-7: human hepatoma-derived
IRE1α: inositol-requiring transmembrane kinase/endoribonuclease 1α
JNK/SAPK: c-JUN NH2-Terminal Kinase/Stress-Activated Protein Kinase
LC3-I & II: Microtubule-associated proteins 1A/1B light chain 3
MA: 3-methyladenine
MAP Kinases: Mitogen-activated protein kinases
mTORC1: Mammalian target of rapamycin
NaF: Sodium fluoride
NF-κB: Nuclear factor kappa-light-chain-enhancer of activated B cells
PARP: Poly (ADP-ribose) polymerase
4-PBA: Sodium phenylbutyrate
PBS: Phosphate buffer saline
PERK: Protein kinase RNA-like endoplasmic reticulum kinase
PI3K: Phosphatidylinositol 3-kinases
PMSF: Phenylmethylsulfonyl fluoride
pRb: Retinoblastoma protein
PVDF: Polyvinylidene fluoride
RIPA: Radio-immunoprecipitation assay
ROS: Reactive oxygen species
SDS-PAGE: Sodium dodecyl sulfate polyacrylamide gel electrophoresis
SEM: Standard error of the mean
SRB: Sulforhodamine B
STZ: Streptozotocin
UPR: Unfolded protein response
XBP1: X-Box binding protein 1
Z-VAD.fmk: carbobenzoxy-valyl-alanyl-aspartyl-[O-methyl]-fluoromethylketone

## References

[1] Noone AM, Howlader N, Krapcho M, Miller D, Brest A, Yu M, Ruhl J, Tatalovich Z, Mariotto A, Lewis DR, Chen HS, Feuer EJ, Cronin KA (eds). SEER Cancer Statistics Review, 1975-2015, National Cancer Institute. Bethesda, MD, https://seer.cancer.gov/csr/1975_2015/, based on November 2017 SEER data submission, posted to the SEER web site, April 2018. https://seer.cancer.gov/archive/csr/1975_2015/.

[2] McGuigan A, Kelly P, Turkington RC, Jones C, Coleman HG, McCain RS (2018). Pancreatic cancer: a review of clinical diagnosis, epidemiology, treatment and outcomes. World J Gastroenterol..

[3] Yabar CS, Winter JM (2016). Pancreatic cancer: a review. Gastroenterol. Clin. N. Am..

[4] Parbhu SK, Adler DG (2016). Pancreatic neuroendocrine tumors: contemporary diagnosis and management. Hosp Pract (1995).

[5] Krug S, Gress TM, Michl P, Rinke A (2017). The role of cytotoxic chemotherapy in advanced pancreatic neuroendocrine tumors. Digestion.

[6] Ito T, Igarashi H, Jensen RT (2012). Pancreatic neuroendocrine tumors: clinical features, diagnosis and medical treatment: advances. Best Pract. Res. Clin. Gastroenterol..

[7] Ro C, Chai W, Yu VE, Yu R (2013). Pancreatic neuroendocrine tumors: biology, diagnosis,and treatment. Chin J Cancer.

[8] Giovannetti E, Mey V, Danesi R, Mosca I, Del Tacca M (2004). Synergistic cytotoxicity and pharmacogenetics of gemcitabine and pemetrexed combination in pancreatic cancer cell lines. Clin. Cancer Res..

[9] Nigri G, Petrucciani N, Debs T, Mangogna LM, Crovetto A, Moschetta G (2018). Treatment options for PNET liver metastases: a systematic review. World J Surgical Oncol.

[10] Gartner S, Kruger J, Aghdassi AA, Steveling A, Simon P, Lerch MM (2016). Nutrition in pancreatic cancer: a review. Gastrointest Tumors.

[11] Li Y, Go VL, Sarkar FH (2015). The role of nutraceuticals in pancreatic cancer prevention and therapy: targeting cellular signaling, MicroRNAs, and epigenome. Pancreas.

[12] Iversen LF, Andersen HS, Branner S, Mortensen SB, Peters GH, Norris K (2000). Structure-based design of a low molecular weight, nonphosphorus, nonpeptide, and highly selective inhibitor of protein-tyrosine phosphatase 1B. J. Biol. Chem..

[13] Bemis DL, Capodice JL, Anastasiadis AG, Katz AE, Buttyan R (2005). Zyflamend, a unique herbal preparation with nonselective COX inhibitory activity, induces apoptosis of prostate cancer cells that lack COX-2 expression. Nutr. Cancer.

[14] Ekmekcioglu S, Chattopadhyay C, Akar U, Gabisi A, Newman RA, Grimm EA (2011). Zyflamend mediates therapeutic induction of autophagy to apoptosis in melanoma cells. Nutr. Cancer.

[15] Yang P, Sun Z, Chan D, Cartwright CA, Vijjeswarapu M, Ding J (2008). Zyflamend reduces LTB4 formation and prevents oral carcinogenesis in a 7,12-dimethylbenz [alpha] anthracene (DMBA)-induced hamster cheek pouch model. Carcinogenesis.

[16] Moore J, Megaly M, MacNeil A, Klentrou P, Tsiani E (2016). Rosemary extract reduces Akt/mTOR/p70S6K activation and inhibits proliferation and survival of A549 human lung cancer cells.

[17] Shakibaei M, Mobasheri A, Lueders C, Busch F, Shayan P, Goel A (2013). Curcumin enhances the effect of chemotherapy against colorectal cancer cells by inhibition of NF-kappaB and Src protein kinase signaling pathways. PLoS One.

[18] Yi Z, Collier JJ, Huang EC, Jay W (2014). Turmeric and Chinese goldthread synergistically inhibit prostate cancer cell proliferation and NF-kB signaling. Functional Foods Health Dis.

[19] MacDonald AF, Bettaieb A, Donohoe DR, Alani DS, Han A, Zhao Y (2018). Concurrent regulation of LKB1 and CaMKK2 in the activation of AMPK in castrate-resistant prostate cancer by a well-defined polyherbal mixture with anticancer properties. BMC Complement. Altern. Med..

[20] Zhao Y, Donohoe D, Huang EC, Whelan J (2015). Zyflamend, a polyherbal mixture, inhibits lipogenesis and mTORC1 signalling via activation of AMPK. J. Funct. Foods.

[21] Subbaramaiah K, Sue E, Bhardwaj P, Du B, Hudis CA, Giri D (2013). Dietary polyphenols suppress elevated levels of proinflammatory mediators and aromatase in the mammary gland of obese mice. Cancer Prev. Res. (Phila.).

[22] Kunnumakkara AB, Sung B, Ravindran J, Diagaradjane P, Deorukhkar A, Dey S (2012). Zyflamend suppresses growth and sensitizes human pancreatic tumors to gemcitabine in an orthotopic mouse model through modulation of multiple targets. Int. J. Cancer.

[23] Kim JH, Park B, Gupta SC, Kannappan R, Sung B, Aggarwal BB (2012). Zyflamend sensitizes tumor cells to TRAIL-induced apoptosis through up-regulation of death receptors and down-regulation of survival proteins: role of ROS-dependent CCAAT/enhancer-binding protein-homologous protein pathway. Antioxid. Redox Signal..

[24] Sandur SK, Ahn KS, Ichikawa H, Sethi G, Shishodia S, Newman RA (2007). Zyflamend, a polyherbal preparation, inhibits invasion, suppresses osteoclastogenesis, and potentiates apoptosis through down-regulation of NF-kappa B activation and NF-kappa B-regulated gene products. Nutr. Cancer.

[25] Xue Y, Yang L, Li J, Yan Y, Jiang Q, Shen L (2018). Combination chemotherapy with Zyflamend reduced the acquired resistance of bladder cancer cells to cisplatin through inhibiting NFkappaB signaling pathway. Onco Targets Ther.

[26] Huang EC, McEntee MF, Whelan J (2012). Zyflamend, a combination of herbal extracts, attenuates tumor growth in murine xenograft models of prostate cancer. Nutr. Cancer.

[27] Souslova T, Averill-Bates DA (2004). Multidrug-resistant hela cells overexpressing MRP1 exhibit sensitivity to cell killing by hyperthermia: interactions with etoposide. Int. J. Radiat. Oncol. Biol. Phys..

[28] Duangjai A, Nuengchamnong N, Suphrom N, Trisat K, Limpeanchob N, Saokaew S (2018). Potential of coffee fruit extract and Quinic acid on Adipogenesis and lipolysis in 3T3-L1 adipocytes. Kobe J Med Sci.

[29] Franken NA, Rodermond HM, Stap J, Haveman J, van Bree C (2006). Clonogenic assay of cells in vitro. Nat. Protoc..

[30] Yan J, Xie B, Capodice JL, Katz AE (2012). Zyflamend inhibits the expression and function of androgen receptor and acts synergistically with bicalutimide to inhibit prostate cancer cell growth. Prostate.

[31] Bettaieb A, Averill-Bates DA (2005). Thermotolerance induced at a mild temperature of 40 degrees C protects cells against heat shock-induced apoptosis. J. Cell. Physiol..

[32] Averill-Bates DA, Cherif A, Agostinelli E, Tanel A, Fortier G (2005). Anti-tumoral effect of native and immobilized bovine serum amine oxidase in a mouse melanoma model. Biochem. Pharmacol..

[33] Cui Q, Yu JH, Wu JN, Tashiro S, Onodera S, Minami M (2007). P53-mediated cell cycle arrest and apoptosis through a caspase-3- independent, but caspase-9-dependent pathway in oridonin-treated MCF-7 human breast cancer cells. Acta Pharmacol. Sin..

[34] Gozzelino R, Sole C, Llecha N, Segura MF, Moubarak RS, Iglesias-Guimarais V (2008). BCL-XL regulates TNF-alpha-mediated cell death independently of NF-kappaB, FLIP and IAPs. Cell Res..

[35] Marciniak SJ, Yun CY, Oyadomari S, Novoa I, Zhang Y, Jungreis R (2004). CHOP induces death by promoting protein synthesis and oxidation in the stressed endoplasmic reticulum. Genes Dev..

[36] Zinszner H, Kuroda M, Wang X, Batchvarova N, Lightfoot RT, Remotti H (1998). CHOP is implicated in programmed cell death in response to impaired function of the endoplasmic reticulum. Genes Dev..

[37] Meley D, Bauvy C, Houben-Weerts JH, Dubbelhuis PF, Helmond MT, Codogno P (2006). AMP-activated protein kinase and the regulation of autophagic proteolysis. J. Biol. Chem..

[38] Wu YT, Tan HL, Shui G, Bauvy C, Huang Q, Wenk MR (2010). Dual role of 3-methyladenine in modulation of autophagy via different temporal patterns of inhibition on class I and III phosphoinositide 3-kinase. J. Biol. Chem..

[39] Shi S, Tan P, Yan B, Gao R, Zhao J, Wang J (2016). ER stress and autophagy are involved in the apoptosis induced by cisplatin in human lung cancer cells. Oncol. Rep..

[40] Wu Z, Wang H, Fang S, Xu C (2018). Roles of endoplasmic reticulum stress and autophagy on H2O2induced oxidative stress injury in HepG2 cells. Mol. Med. Rep..

[41] Zhang L, Cheng X, Xu S, Bao J, Yu H (2018). Curcumin induces endoplasmic reticulum stress-associated apoptosis in human papillary thyroid carcinoma BCPAP cells via disruption of intracellular calcium homeostasis. Medicine (Baltimore).

[42] Li H, Chen H, Li R, Xin J, Wu S, Lan J, Xue K, Li X, Zuo C, Jiang W, Zhu L. Cucurbitacin I induces cancer cell death through the endoplasmic reticulum stress pathway. J Cell Biochem. 2019;120:2391–403. 10.1002/jcb.27570 30277611

[43] Matsuzawa A, Nishitoh H, Tobiume K, Takeda K, Ichijo H (2002). Physiological roles of ASK1-mediated signal transduction in oxidative stress- and endoplasmic reticulum stress-induced apoptosis: advanced findings from ASK1 knockout mice. Antioxid. Redox Signal..

[44] Nishitoh H, Saitoh M, Mochida Y, Takeda K, Nakano H, Rothe M (1998). ASK1 is essential for JNK/SAPK activation by TRAF2. Mol. Cell.

[45] Orzechowska EJ, Girstun A, Staron K, Trzcinska-Danielewicz J (2015). Synergy of BID with doxorubicin in the killing of cancer cells. Oncol. Rep..

[46] Siedlakowski P, McLachlan-Burgess A, Griffin C, Tirumalai SS, McNulty J, Pandey S (2008). Synergy of Pancratistatin and tamoxifen on breast cancer cells in inducing apoptosis by targeting mitochondria. Cancer Biol Ther.

[47] Zhu J, Chen Y, Ji Y, Yu Y, Jin Y, Zhang X (2018). Gemcitabine induces apoptosis and autophagy via the AMPK/mTOR signaling pathway in pancreatic cancer cells. Biotechnol. Appl. Biochem..

[48] Zhang L, Fang Y, Xu XF, Jin DY (2017). Moscatilin induces apoptosis of pancreatic cancer cells via reactive oxygen species and the JNK/SAPK pathway. Mol. Med. Rep..

[49] Zhang P, Lai ZL, Chen HF, Zhang M, Wang A, Jia T (2017). Curcumin synergizes with 5-fluorouracil by impairing AMPK/ULK1-dependent autophagy, AKT activity and enhancing apoptosis in colon cancer cells with tumor growth inhibition in xenograft mice. J. Exp. Clin. Cancer Res..

[50] Maheshwari RK, Singh AK, Gaddipati J, Srimal RC (2006). Multiple biological activities of curcumin: a short review. Life Sci..

[51] Beevers CS, Li F, Liu L, Huang S (2006). Curcumin inhibits the mammalian target of rapamycin-mediated signaling pathways in cancer cells. Int. J. Cancer.

[52] Huang S (2013). Inhibition of PI3K/Akt/mTOR signaling by natural products. Anti Cancer Agents Med. Chem..

[53] Shankar S, Srivastava RK (2007). Involvement of Bcl-2 family members, phosphatidylinositol 3′-kinase/AKT and mitochondrial p53 in curcumin (diferulolylmethane)-induced apoptosis in prostate cancer. Int. J. Oncol..

[54] Yoshida K, Toden S, Ravindranathan P, Han H, Goel A (2017). Curcumin sensitizes pancreatic cancer cells to gemcitabine by attenuating PRC2 subunit EZH2, and the lncRNA PVT1 expression. Carcinogenesis.

[55] Pashaei-Asl R, Pashaei-Asl F, Mostafa Gharabaghi P, Khodadadi K, Ebrahimi M, Ebrahimie E (2017). The inhibitory effect of ginger extract on ovarian cancer cell line; application of systems biology. Adv Pharm Bull.

[56] Prasad S, Tyagi AK (2015). Ginger and its constituents: role in prevention and treatment of gastrointestinal cancer. Gastroenterol. Res. Pract..

[57] Lee SH, Cekanova M, Baek SJ (2008). Multiple mechanisms are involved in 6-gingerol-induced cell growth arrest and apoptosis in human colorectal cancer cells. Mol. Carcinog..

[58] Kim SO, Kim MR (2013). [6]-Gingerol prevents disassembly of cell junctions and activities of MMPs in invasive human pancreas cancer cells through ERK/NF- kappa B/snail signal transduction pathway. Evid. Based Complement. Alternat. Med..

[59] Shamoto T, Matsuo Y, Shibata T, Tsuboi K, Nagasaki T, Takahashi H (2014). Zerumbone inhibits angiogenesis by blocking NF-kappaB activity in pancreatic cancer. Pancreas.

[60] Zhou L, Qi L, Jiang L, Zhou P, Ma J, Xu X (2014). Antitumor activity of gemcitabine can be potentiated in pancreatic cancer through modulation of TLR4/NF-kappaB signaling by 6-shogaol. AAPS J..

[61] Moore J, Yousef M, Tsiani E (2016). Anticancer effects of rosemary (*Rosmarinus officinalis* L.) extract and rosemary extract polyphenols. Nutrients.

[62] Kontogianni VG, Tomic G, Nikolic I, Nerantzaki AA, Sayyad N, Stosic-Grujicic S (2013). Phytochemical profile of Rosmarinus officinalis and Salvia officinalis extracts and correlation to their antioxidant and anti-proliferative activity. Food Chem..

[63] Cheung SSC, Tai J, Hasman D, Ou D, Warnock GL (2015). Inhibition of human pancreatic cancer cell proliferation by Devil's Club Oplopanax horridus and its Polyacetylene bioactive compound. Nutr. Cancer.

[64] González-Vallinas M, Molina S, Vicente G, Zarza V, Martín-Hernández R, García-Risco MR (2014). Expression of microRNA-15b and the glycosyltransferase GCNT3 correlates with antitumor efficacy of rosemary diterpenes in colon and pancreatic cancer. PLoS One.

[65] Park YJ, Wen J, Bang S, Park SW, Song SY (2006). [6]-Gingerol induces cell cycle arrest and cell death of mutant p53-expressing pancreatic cancer cells. Yonsei Med. J..

[66] Zhang S, Liu Q, Liu Y, Qiao H, Liu Y (2012). Zerumbone, a southeast Asian ginger Sesquiterpene, induced apoptosis of pancreatic carcinoma cells through p53 signaling pathway. Evid. Based Complement. Alternat. Med..

[67] Gonzalez-Vallinas M, Molina S, Vicente G, Sanchez-Martinez R, Vargas T, Garcia-Risco MR (2014). Modulation of estrogen and epidermal growth factor receptors by rosemary extract in breast cancer cells. Electrophoresis.

[68] Petiwala SM, Berhe S, Li G, Puthenveetil AG, Rahman O, Nonn L (2014). Rosemary (Rosmarinus officinalis) extract modulates CHOP/GADD153 to promote androgen receptor degradation and decreases xenograft tumor growth. PLoS One.

[69] Shimizu T, Torres MP, Chakraborty S, Souchek JJ, Rachagani S, Kaur S (2013). Holy basil leaf extract decreases tumorigenicity and metastasis of aggressive human pancreatic cancer cells in vitro and in vivo: potential role in therapy. Cancer Lett..

[70] Huang EC, Chen G, Baek SJ, McEntee MF, Collier JJ, Minkin S (2011). Zyflamend reduces the expression of androgen receptor in a model of castrate-resistant prostate cancer. Nutr. Cancer.

[71] Jatoi A, Ellison N, Burch PA, Sloan JA, Dakhil SR, Novotny P (2003). A phase II trial of green tea in the treatment of patients with androgen independent metastatic prostate carcinoma. Cancer.

[72] Guertin DA, Sabatini DM (2007). Defining the role of mTOR in cancer. Cancer Cell.

[73] Tyagi N, Bhardwaj A, Singh AP, McClellan S, Carter JE, Singh S (2014). P-21 activated kinase 4 promotes proliferation and survival of pancreatic cancer cells through AKT- and ERK-dependent activation of NF-kappaB pathway. Oncotarget.

[74] Hung JY, Hsu YL, Li CT, Ko YC, Ni WC, Huang MS (2009). 6-Shogaol, an active constituent of dietary ginger, induces autophagy by inhibiting the AKT/mTOR pathway in human non-small cell lung cancer A549 cells. J. Agric. Food Chem..

[75] Zhao Z, Li C, Xi H, Gao Y, Xu D (2015). Curcumin induces apoptosis in pancreatic cancer cells through the induction of forkhead box O1 and inhibition of the PI3K/Akt pathway. Mol. Med. Rep..

[76] Fujita E, Kouroku Y, Isoai A, Kumagai H, Misutani A, Matsuda C (2007). Two endoplasmic reticulum-associated degradation (ERAD) systems for the novel variant of the mutant dysferlin: ubiquitin/proteasome ERAD(I) and autophagy/lysosome ERAD (II). Hum. Mol. Genet..

[77] Rashid HO, Yadav RK, Kim HR, Chae HJ (2015). ER stress: autophagy induction, inhibition and selection. Autophagy.

[78] Kondo Y, Kanzawa T, Sawaya R, Kondo S (2005). The role of autophagy in cancer development and response to therapy. Nat. Rev. Cancer.

[79] Verfaillie T, Salazar M, Velasco G, Agostinis P (2010). Linking ER stress to autophagy: potential implications for cancer therapy. Int J Cell Biol.

[80] Klieser E, Illig R, Stattner S, Primavesi F, Jager T, Swierczynski S (2015). Endoplasmic reticulum stress in pancreatic neuroendocrine tumors is linked to Clinicopathological parameters and possible epigenetic regulations. Anticancer Res..

[81] Salazar M, Carracedo A, Salanueva IJ, Hernandez-Tiedra S, Lorente M, Egia A (2009). Cannabinoid action induces autophagy-mediated cell death through stimulation of ER stress in human glioma cells. J. Clin. Invest..

[82] Huang EC, Zhao Y, Chen G, Baek SJ, McEntee MF, Minkin S (2014). Zyflamend, a polyherbal mixture, down regulates class I and class II histone deacetylases and increases p21 levels in castrate-resistant prostate cancer cells. BMC Complement. Altern. Med..

