# Supplementary material for: Zyflamend induces apoptosis in pancreatic cancer cells via modulation of the JNK pathway
Source: Cell Commun Signal. 2020 Aug 14;18:126. doi: 10.1186/s12964-020-00609-7 (PMC7427957; doi:10.1186/s12964-020-00609-7)
Supplement: Supplementary file 2 — Additional file 1: Supplementary Table 1. List of Primary Antibodies and Conditions of Use. Supplementary Table 2. Composition of Zyflamend [82]. [file 12964_2020_609_MOESM1_ESM.docx]

**Supplementary** **Table 1: List of Primary Antibodies and Conditions of Use.**

| **Antibodies** | **Source** | **Catalog Number** | **MW (kDa)** | **Host** | **Dilution** |
| --- | --- | --- | --- | --- | --- |
| AKT | Cell Signaling Technology | 2920 | 60 | Rabbit | 1:5,000 |
| AMPK | Cell Signaling Technology | 5832 | 62 | Rabbit | 1:5,000 |
| ATG5 | Cell Signaling Technology | 12994 | 55 | Rabbit | 1:1,000 |
| ATG7 | Cell Signaling Technology | 8558 | 80 | Rabbit | 1:1,000 |
| Beclin 1 | Santa Cruz Biotechnology | sc-48381 | 60 | Mouse | 1:1,000 |
| CHOP | Santa Cruz Biotechnology | sc-7351 | 29 | Mouse | 1:5,000 |
| Cleaved Caspase-3 | Cell Signaling Technology | 9662 | 17 | Rabbit | 1:5,000 |
| EIF2α | Santa Cruz Biotechnology | sc-1386 | 38 | Mouse | 1:1,000 |
| ERK1/2 | Cell Signaling Technology | 4695 | 42/44 | Rabbit | 1:5,000 |
| IRE1 | Cell Signaling Technology | 3294 | 115 | Rabbit | 1:1,000 |
| JNK1/2 | Santa Cruz Biotechnology | sc-7345 | 46/54 | Mouse | 1:1,000 |
| LC3-I/II | Bio-Rad | AHP2167 | 13/15 | Rabbit | 1:250 |
| P38 | Santa Cruz Biotechnology | sc-7972 | 42 | Mouse | 1:1,000 |
| PARP | Santa Cruz Biotechnology | sc-53643 | 115/90 | Rabbit | 1:1,000 |
| PERK | Cell Signaling Technology | 3192 | 130 | Rabbit | 1:1,000 |
| Phospho-AKT ^S473^ | Cell Signaling Technology | 3527 | 60 | Rabbit | 1:2,500 |
| Phospho-AMPK^T172^ | Cell Signaling Technology | 2535 | 62 | Rabbit | 1:2,500 |
| Phospho-EIF2α^S51^ | Santa Cruz Biotechnology | sc- 293100 | 38 | Rabbit | 1:1,000 |
| Phospho-ERK1/2 ^T202/Y204^ | Cell Signaling Technology | 9101 | 42/44 | Rabbit | 1:5,000 |
| Phospho-IRE1^S724^ | Abcam | ab 48187 | 115 | Rabbit | 1:10,000 |
| Phospho-JNK1/2^T183/Y185^ | Santa Cruz Biotechnology | sc-6254 | 46/54 | Mouse | 1:1,000 |
| Phospho-P38^T180/Y182^ | Cell Signaling Technology | 4511 | 42 | Mouse | 1:10,000 |
| Phospho-PERK^T980^ | Santa Cruz Biotechnology | sc-32577 | 160 | Rabbit | 1:1,000 |
| sXBP1 | Santa Cruz Biotechnology | sc-32136 | 58 | Goat | 1:500 |
| β-actin | Santa Cruz Biotechnology | sc-47778 | 44 | Mouse | 1:20,000 |

**Supplementary Table 2. Composition of Zyflamend [81].**

| **Extract** | **Latin Name** | **Amount (%)** |
| --- | --- | --- |
| Rosemary | Rosmarinus officinalis | 19.2 |
| Turmeric | Curcuma longa | 14.1 |
| Ginger | Zingiber officinale | 12.8 |
| Holy Basil | Ocimum sanctum | 12.8 |
| Organic Green Tea | Camellia sinensis | 12.8 |
| Hu Zhang | Polygonum cuspidatum | 10.2 |
| Chinese Goldthread | Coptis chinensis | 5.1 |
| Barberry | Berberis vulgaris | 5.1 |
| Organic Oregano | Origanum vulgare | 5.1 |
| Chinese Skullcap | Scutellaria baicalensis | 2.5 |
