# Supplementary figures and images for: Zyflamend induces apoptosis in pancreatic cancer cells via modulation of the JNK pathway
Source: Cell Commun Signal. 2020 Aug 14;18:126. doi: 10.1186/s12964-020-00609-7 (PMC7427957; doi:10.1186/s12964-020-00609-7)

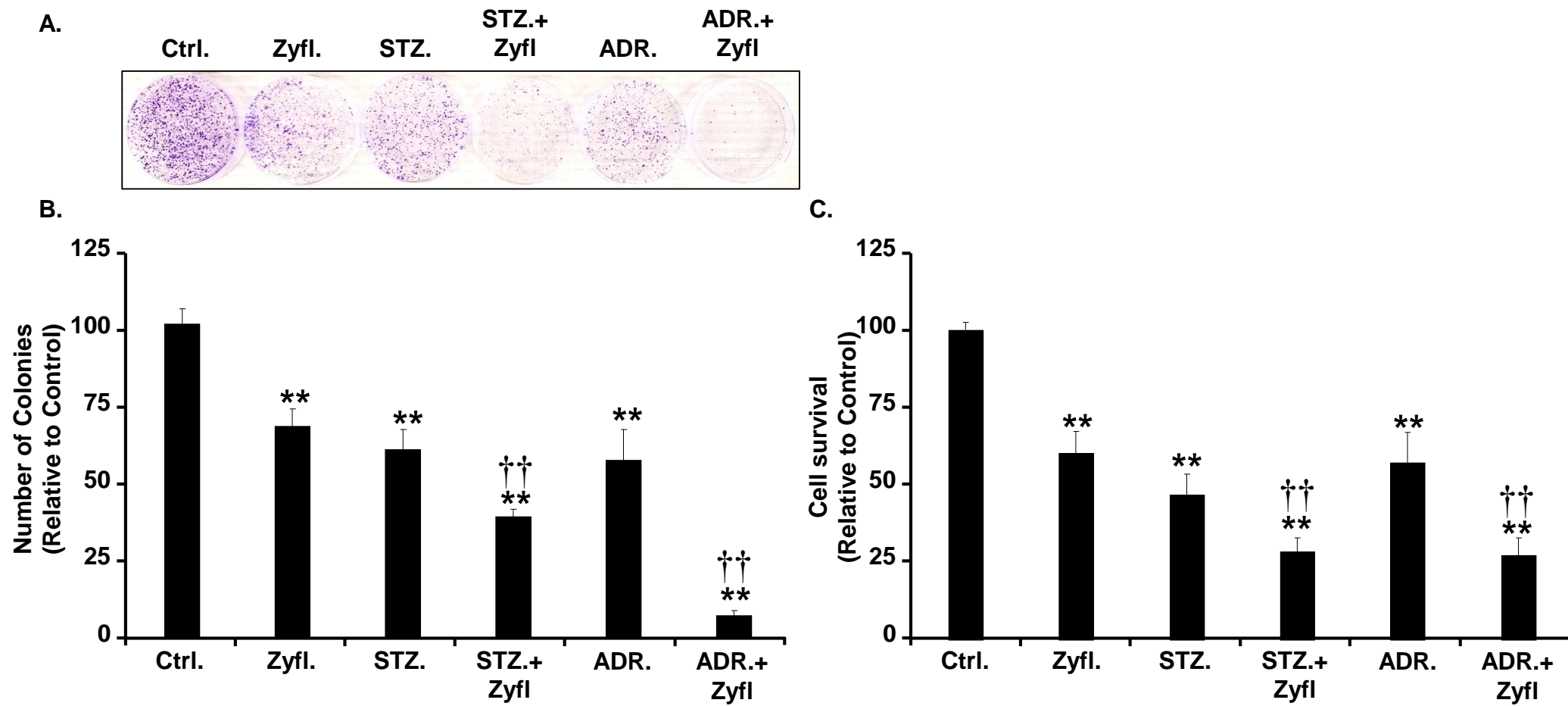

Supplementary Figure S1

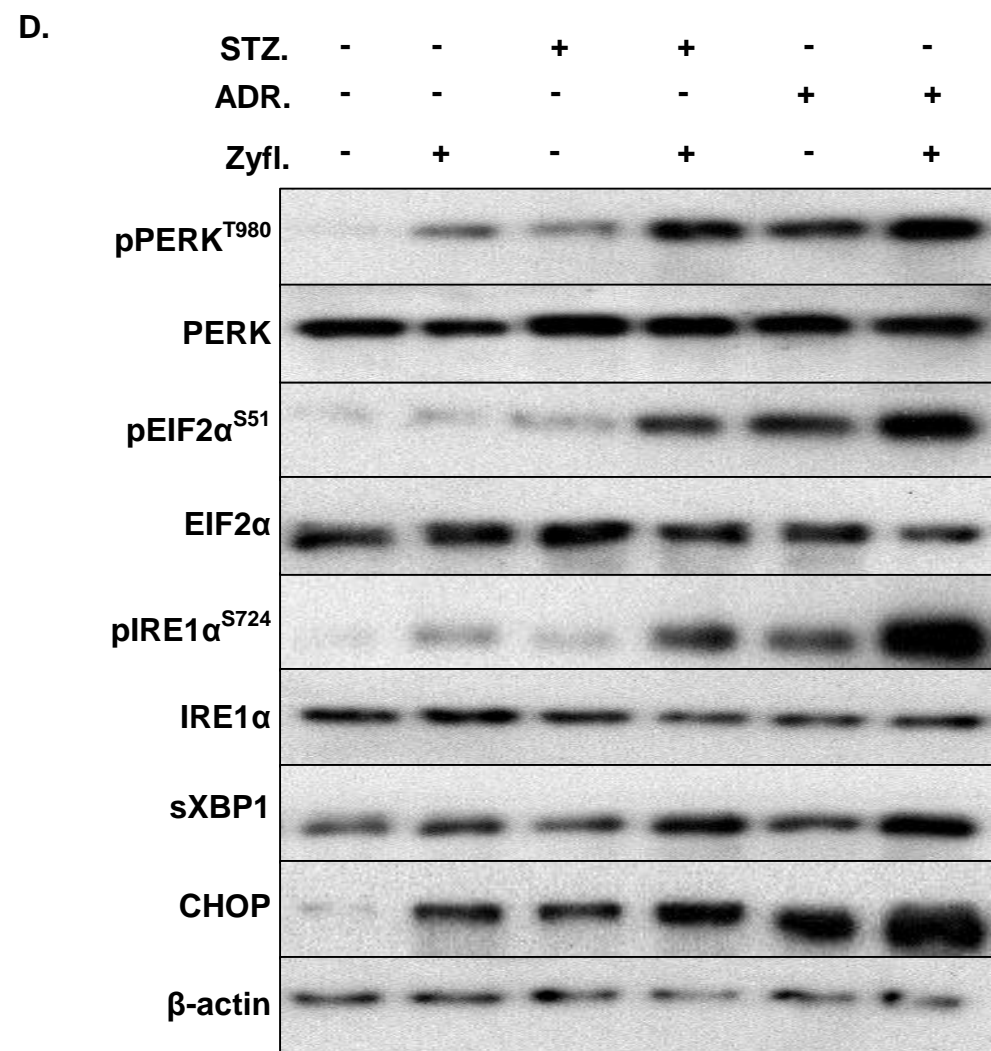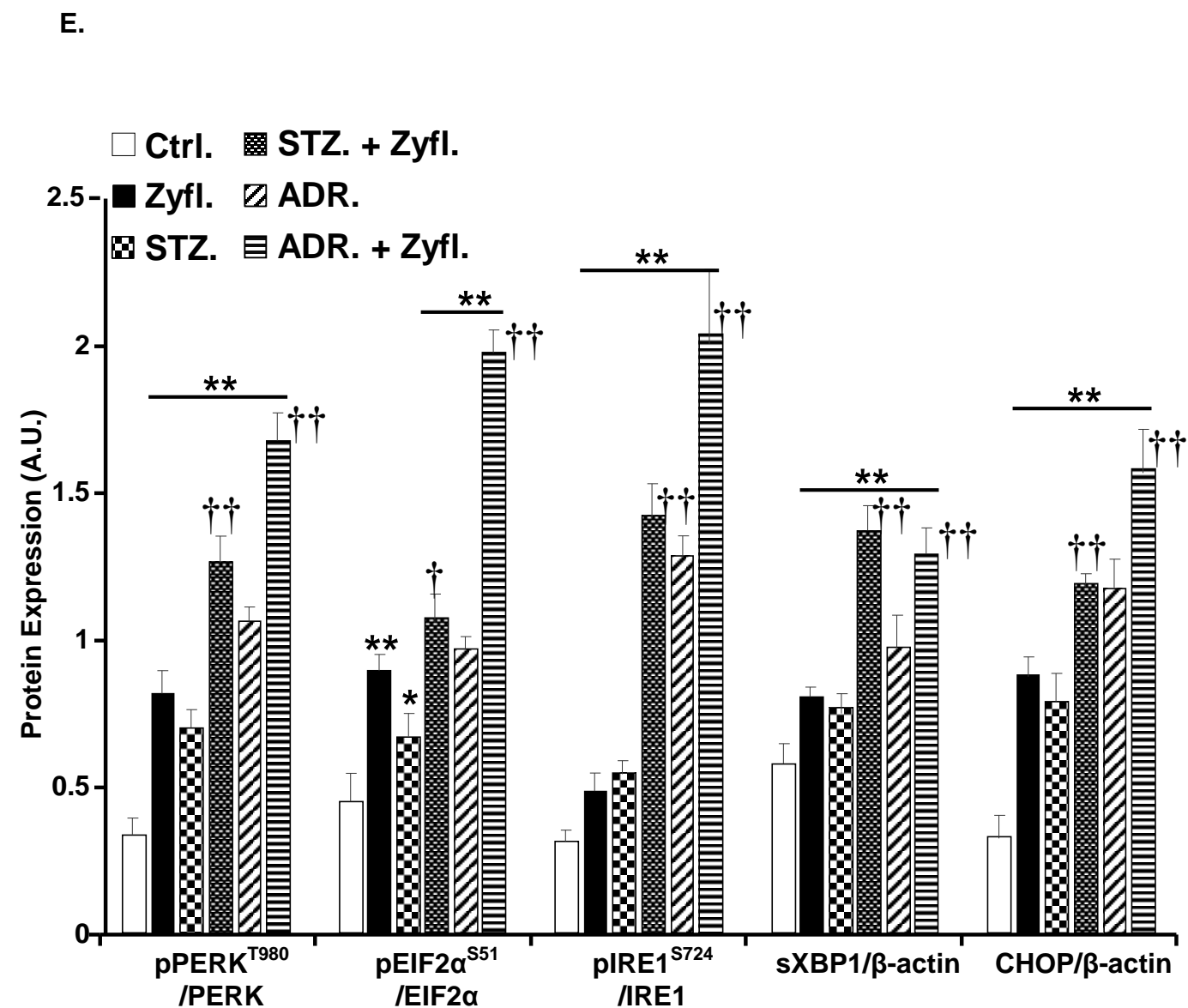

Supplementary Figure S1

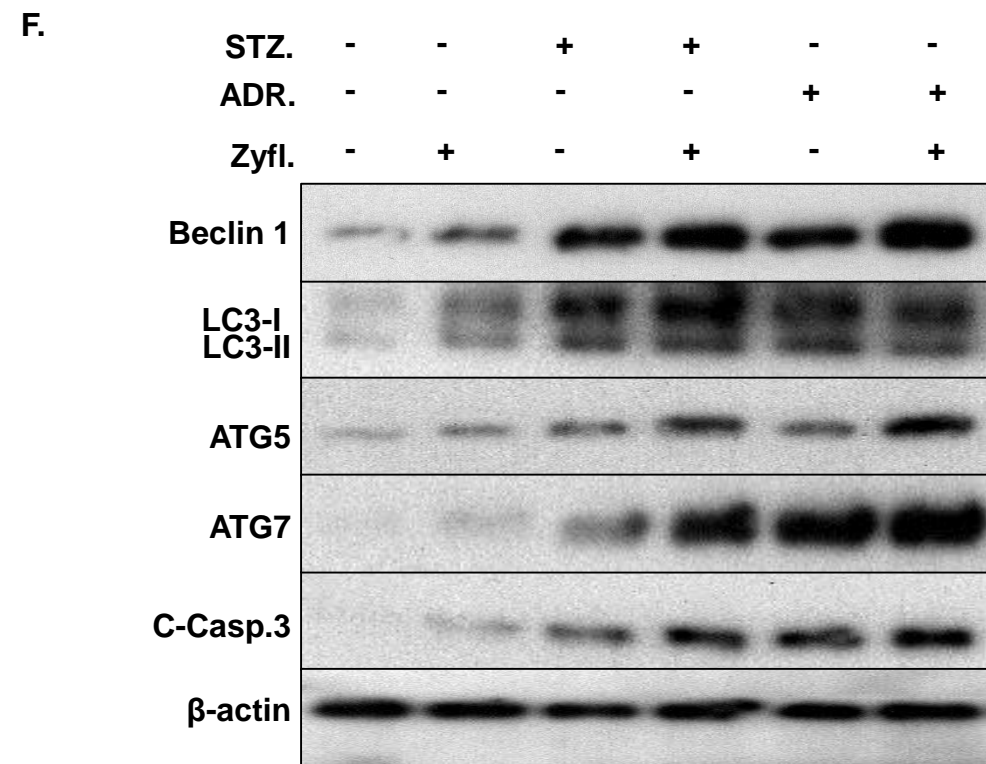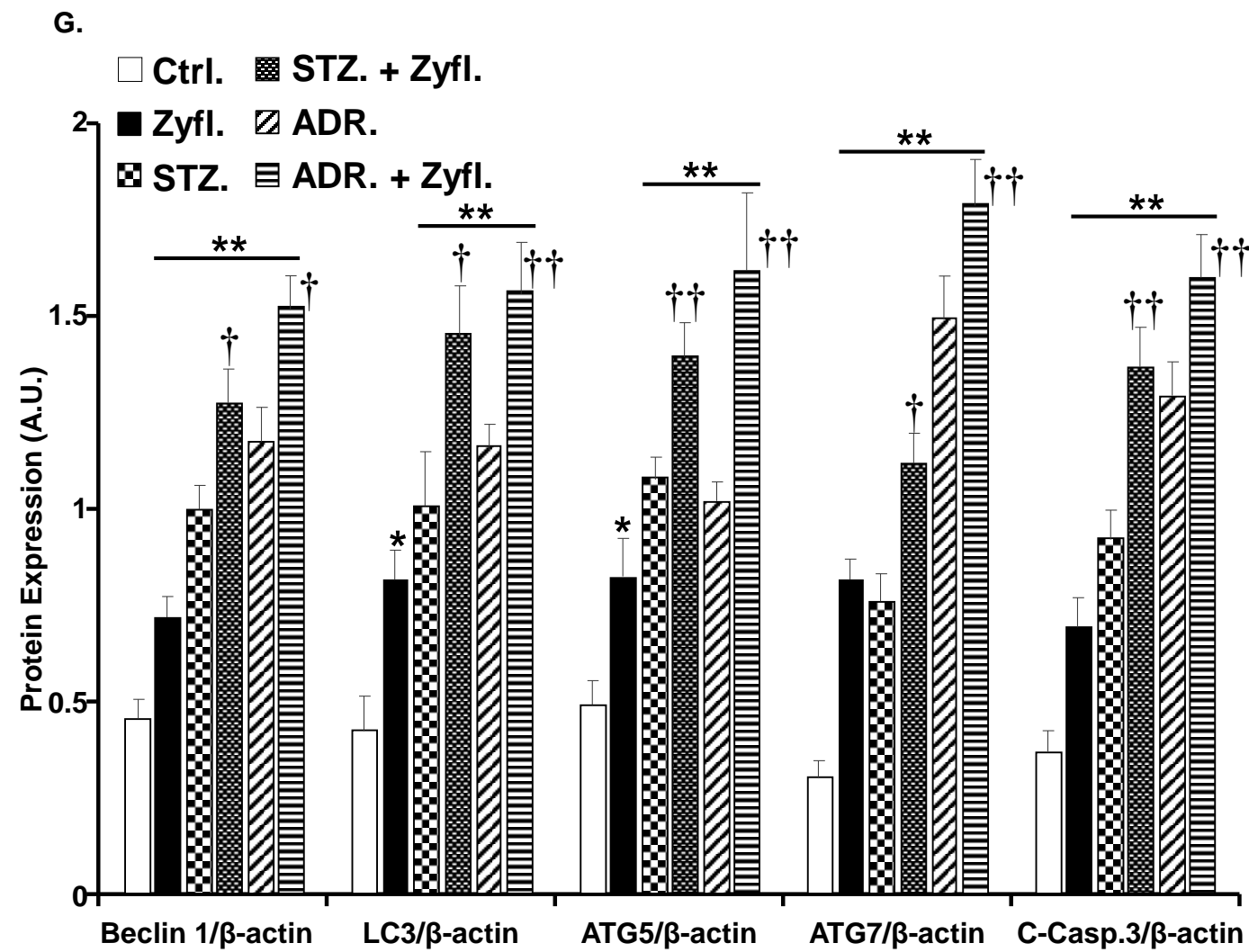

Supplementary Figure S1

Supplement: Supplementary file 3 — Additional file 2: Supplementary Fig. 1. Zyflamend Causes Toxicity and Exacerbates the Effects of Streptozotocin and Adriamycin on ER Stress, Autophagy, and Cell Death in RIN-5F Cells. A-B) Colony formation assay. B) Bar graphs represent the relative number of colonies in each condition determined by dividing the number of colonies for a given treatment by the total number of colonies in DMSO treated cells (Ctrl.) and expressed as a percentage. C) Cell toxicity assay using the MTT method. Bar graphs represent the intensity of formazan staining reflective of the cell number and presented as means + SEM. D-G) Total cell lysates from control cells treated with Zyflamend and non-treated cells with or without streptozotocin (STZ; 5 mM) or adriamycin (ADR; 5 μM) for 24 h were immunoblotted for markers of ER stress (D-E), autophagy, and cell death (F-G). Representative immunoblots are shown. E) Bar graphs represent pPERK/PERK, pEIF2α/EIF2α, pIRE1/IRE1, sXBP1/β-actin, and CHOP/β-actin as means + SEM. *p < 0.05, **p < 0.01 indicate a significant difference between cells treated with Zyflamend and non-treated cells. G) Bar graphs represent Beclin 1/β-actin, LC3/β-actin, ATG5/β-actin, ATG7/β-actin, and cleaved caspase-3 / β-actin as means + SEM. In C, E, and G *p < 0.05, **p < 0.01 indicate a significant difference between cells treated with Zyflamend and non-treated cells. †p < 0.05, ††p < 0.01 indicate a significant difference between cells treated with Zyflamend combined with the chemotherapeutic agents (streptozotocin or adriamycin) and cells treated with streptozotocin or adriamycin only. [file 12964_2020_609_MOESM2_ESM.pdf]
